# Omics Profiling of S2P Mutant Fibroblasts as a Mean to Unravel the Pathomechanism and Molecular Signatures of X-Linked *MBTPS2* Osteogenesis Imperfecta

**DOI:** 10.3389/fgene.2021.662751

**Published:** 2021-05-21

**Authors:** Pei Jin Lim, Severin Marfurt, Uschi Lindert, Lennart Opitz, Timothée Ndarugendamwo, Pakeerathan Srikanthan, Martin Poms, Martin Hersberger, Claus-Dieter Langhans, Dorothea Haas, Marianne Rohrbach, Cecilia Giunta

**Affiliations:** ^1^Connective Tissue Unit, Division of Metabolism and Children’s Research Centre, University Children’s Hospital, Zurich, Switzerland; ^2^University of Zürich, Zurich, Switzerland; ^3^Functional Genomics Center Zurich, University of Zurich/ETH Zurich, Zurich, Switzerland; ^4^Division of Clinical Chemistry and Biochemistry, University Children’s Hospital Zurich, Zurich, Switzerland; ^5^Department of Pediatrics, Centre for Pediatric and Adolescent Medicine, Division of Neuropediatrics and Metabolic Medicine, University Hospital, Heidelberg, Germany

**Keywords:** X-linked osteogenesis imperfecta, connective tissue, transcriptomics, *MBTPS2*, site 2 protease

## Abstract

Osteogenesis imperfecta (OI) is an inherited skeletal dysplasia characterized by low bone density, bone fragility and recurrent fractures. The characterization of its heterogeneous genetic basis has allowed the identification of novel players in bone development. In 2016, we described the first X-linked recessive form of OI caused by hemizygous *MBTPS2* missense variants resulting in moderate to severe phenotypes. *MBTPS2* encodes site-2 protease (S2P), which activates transcription factors involved in bone (OASIS) and cartilage development (BBF2H7), ER stress response (ATF6) and lipid metabolism (SREBP) via regulated intramembrane proteolysis. In times of ER stress or sterol deficiency, the aforementioned transcription factors are sequentially cleaved by site-1 protease (S1P) and S2P. Their N-terminal fragments shuttle to the nucleus to activate gene transcription. Intriguingly, missense mutations at other positions of *MBTPS2* cause the dermatological spectrum condition Ichthyosis Follicularis, Atrichia and Photophobia (IFAP) and Keratosis Follicularis Spinulosa Decalvans (KFSD) without clinical overlap with OI despite the proximity of some of the pathogenic variants. To understand how single amino acid substitutions in S2P can lead to non-overlapping phenotypes, we aimed to compare the molecular features of *MBTPS2*-OI and *MBTPS2*-IFAP/KFSD, with the ultimate goal to unravel the pathomechanisms underlying *MBTPS2*-OI. RNA-sequencing-based transcriptome profiling of primary skin fibroblasts from healthy controls (*n* = 4), *MBTPS2*-OI (*n* = 3), and *MBTPS2-*IFAP/KFSD (*n* = 2) patients was performed to identify genes that are differentially expressed in *MBTPS2*-OI and *MBTPS2-*IFAP/KFSD individuals compared to controls. We observed that SREBP-dependent genes are more downregulated in OI than in IFAP/KFSD. This is coupled to alterations in the relative abundance of fatty acids in *MBTPS2*-OI fibroblasts *in vitro*, while no consistent alterations in the sterol profile were observed. Few OASIS-dependent genes are suppressed in *MBTPS2*-OI, while BBF2H7- and ATF6-dependent genes are comparable between OI and IFAP/KFSD patients and control fibroblasts. Importantly, we identified genes involved in cartilage physiology that are differentially expressed in *MBTPS2-*OI but not in *MBTPS2*-IFAP/KFSD fibroblasts. In conclusion, our data provide clues to how pathogenic *MBTPS2* mutations cause skeletal deformities via altered fatty acid metabolism or cartilage development that may affect bone development, mineralization and endochondral ossification.

## Introduction

Osteogenesis imperfecta (OI), known more commonly as brittle bone disease, is a genetically heterogeneous disorder of bone matrix formation and remodeling and represents one of the most common form of skeletal dysplasia (Krakow, 2015). OI is characterized by low bone density, recurrent fractures, bone deformities and short stature. Its additional features which include dentinogenesis imperfecta, progressive hearing loss and blue-gray hue of the sclerae demonstrate generalized connective tissue disorder and highlight the systemic nature of the disease presentation (Marini et al., 2017). The spectrum of severity of the clinical presentation of OI is broad, ranging from mild to severe forms with perinatal lethality. There is currently no cure for OI due to a yet incomplete understanding of its underlying pathomechanisms. The management of patients currently consists of a combination of supportive rehabilitation, orthopedic interventions to correct bone and joint deformities and pain management of acute fractures. The inhibition of bone resorption by intravenous or oral administration of bisphosphonates has become the mostly used pharmacological treatment in children and adults with OI who benefit from reduced bone turnover, higher bone mineral density (BMD) and lower fracture rate (DiMeglio et al., 2005; Shapiro et al., 2010).

Of the known causative genes, *MBTPS2* (reference sequences NG_012797.2, NM_015884.4) was recently characterized as the first X-linked cause of OI (OMIM 301014). In a cohort of affected males from two unrelated families (p.Asn459Ser in a Thai family and p.Leu505Phe in a German family), we identified a classical OI phenotype with moderate severity (Lindert et al., 2016). A third family which was recently characterized indicated a possible lethal intrauterine effect caused by the p.Glu172Asp variant (Dr. Jason Pinner, personal communication), suggesting that mutations in *MBTPS2* might cause the full clinical severity spectrum of OI. The initial characterization of *MBTPS2*-OI revealed new insights into molecular mechanisms that are potentially relevant for bone homeostasis.

*MBTPS2* encodes the Golgi resident protein site-2 protease (S2P). S2P is a zinc metalloprotease involved in regulated intramembrane proteolysis (RIP), a process occurring on the Golgi membrane that activates several membrane-bound transcription factors. These include activating transcription factor 6 (ATF6), old astrocyte specifically induced substance (OASIS), box B-binding factor 2 human homolog on chromosome 7 (BBF2H7) and sterol regulatory element binding protein (SREBP) (Rawson, 2013). Upon RIP cleavage by S2P, the activated transcription factors that are released from the Golgi membrane translocate into the nucleus, where they exert their regulatory effects on the expression of genes involved in Endoplasmic Reticulum (ER) stress response (Ye et al., 2000), osteoblast differentiation (Murakami et al., 2009), chondrogenesis (Saito et al., 2009; Izumi et al., 2012), and lipid homeostasis (Horton et al., 2003), respectively. Notably, the activities of these transcription factors are induced by the RIP process when cells undergo ER stress (Ye et al., 2000; Murakami et al., 2006; Saito et al., 2009; Izumi et al., 2012; Zhang et al., 2012a; Rawson, 2013).

**FIGURE 1:**
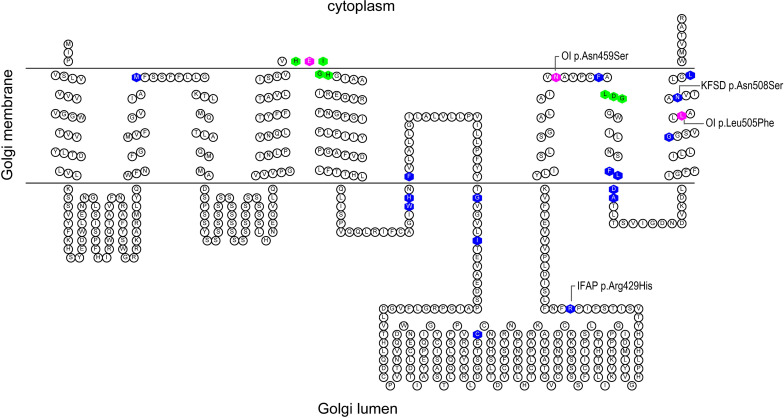
Predicted secondary structure of S2P based on Uniprot topology annotations. The figure was generated using the TOPO2 display software for transmembrane proteins (Johns, 2020). The histidine residues of the His-Glu-(any amino acid)_2_-His (H_171_EXXH) motif and aspartate residue of the L_466_DG sequence, depicted in green, coordinate the catalytic site zinc atom of S2P. Known IFAP/KFSD-causative variants are depicted in blue; OI-causative variants are depicted in magenta.

Intriguingly, missense mutations at different positions of S2P cause distinct syndromes. Prior to its association with OI, pathogenic missense variants in *MBTPS2* were described in the dermatological spectrum condition Ichthyosis Follicularis, Atrichia, and Photophobia (IFAP, OMIM 308205) and Keratosis Follicularis Spinulosa Decalvans (KFSD, OMIM 308800) (Oeffner et al., 2009; Aten et al., 2010; Bornholdt et al., 2013). *MBTPS2* mutations causing IFAP/KFSD and OI may be located in proximity according to a predicted secondary structure of S2P (Figure 1) despite the lack of clinical overlap. This offers a unique biological context to explore the pathophysiology in the skeletal system by identifying mechanisms and molecules that are specifically perturbed in *MBTPS2*-OI but not in IFAP/KFSD and might be relevant to the development of bone and cartilage.

We previously showed that mutant S2P proteins are stably expressed in OI and IFAP/KFSD patient-derived fibroblasts, but cleavage of OASIS is impaired. Furthermore, co-transfection of mutant or wildtype *MBTPS2* constructs and luciferase under the control of either ATF6 or SREBP in CHO-M19 cells lacking endogenous S2P demonstrated diminished ATF6 and SREBP activities upon ER stress induction in the cells expressing mutant S2P proteins. The data collectively suggest that S2P pathogenic variants in OI and IFAP/KFSD cause defective RIP and activation of the transcription factors (Lindert et al., 2016).

In this follow-up study, we aim to further characterize the molecular features of *MBTPS2*-OI and compare it to that of IFAP/KFSD caused by *MBTPS2* mutations (*MBTPS2*-IFAP/KFSD) and healthy controls. In particular, we investigate whether molecular changes occur uniquely in response to pathogenic *MBTPS2* variants causative of OI but not in response to variants causative of IFAP/KFSD, and whether these unique changes provide insights into mechanisms associated with skeletal dyshomeostasis. To this end, we performed transcriptome profiling of primary skin fibroblasts, since S2P is involved in the RIP-regulated activation of the aforementioned transcription factors. Lipid analyses, electron microscopy and immunofluorescence assay were subsequently performed for the functional validation of the transcriptomics data.

## Materials and Methods

### Subjects and Cell Culture

Punch skin biopsies were obtained from OI and IFAP/KFSD patients with pathogenic *MBTPS2* variants to establish primary skin fibroblasts cultures. The biopsies of three *MBTPS2*-OI patients (two patients with c.1515**G**>**C**; p.(Leu505Phe) and one patient with c.1376**A**>**G**; p.(Asn459Ser)), two *MBTPS2*-IFAP/KFSD patients (c.1286**G**>**A**; p.(Arg429His) and c.1523**A**>**G**; p.(Asn508Ser)) and four healthy controls were used for this study (Supplementary Table 1). Informed consent of the patients or their parents, in accordance with requirements of the local ethics committees of the referring physicians, was obtained. This study was conducted according to the Declaration of Helsinki and approved by Swiss Ethics Committee (KEK-ZH-Nr. 2019-00811).

Cells were cultured at 37°C and 5% CO_2_ in Dulbecco’s Modified Eagle’s Medium (Gibco, 31966-021) supplemented with 10% fetal bovine serum (Gibco, 10270-106), and antibiotic-antimycotic (Gibco, 15240-06215240062) compromising of 100 U/ml penicillin, 100 μg/ml streptomycin, and 0.25 μg/ml Amphotericin B. Passage numbers of the cells used throughout all experiments in this study ranged between P8 and P17.

### Gene Expression Profiling

Fibroblasts were passage into T75 flasks, fed fresh medium in the absence or presence of 500 nM thapsigargin (Sigma, T9033) 24 h after passaging, and RNA was isolated 20 h later without synchronization for transcriptome profiling. RNA was harvested using miRNeasy Mini Kit (QIAGEN, 217004) according to the manufacturer’s instructions. RNA quality control was performed on an Agilent 2100 Bioanalyzer with RNA integrity number (RIN) values between 9.6 and 10.0. Poly-A purified libraries were prepared using the TruSeq mRNA sample preparation kit (Illumina, 20020595). RNA-sequencing was performed on an Illumina HiSeq 4000 instrument at the Functional Genomics Center Zurich. The raw reads were cleaned by removing adapter sequences, trimming low quality ends, and filtering reads with low quality (phred quality < 20). Sequence alignment of the resulting high-quality reads to the human genome (build GRCh38.p10) and quantification of transcript expression was carried out using RSEM (version 1.3.0, RRID: SCR_013027) with Ensembl gene models of release 89. A count based negative binomial model implemented in the software package edgeR (R-version 3.5.1, edgeR 3.24.2, RRID: SCR_012802) was applied to detect differentially expressed genes (DEGs). Principal Component Analyses (PCA) were performed using the prcomp function in the R package “stats.” The RNA-sequencing data, including raw sequence files for each subject, have been uploaded onto the European Nucleotide Archive (ENA) under the accession ID PRJEB42767.

Differentially expressed genes were defined as those whose expression level differed between patient and control groups with *p* < 0.05 and log_2_(fold change) > 0.5 or < −0.5. This definition was used for the generation of volcano plots for visual representation of the RNA-sequencing dataset.

To identify biological processes, cellular components and molecular functions that are over-represented by DEGs in *MBTPS2*-OI and *MBTPS2*-IFAP/KFSD, genes whose expression levels differed between patient and control groups with *p* < 0.01 and log_2_(fold change) > 0.5 or < −0.5 were subjected to over-representation analysis (ORA) using the WebGestalt bioinformatics toolkit (Zhang et al., 2005; Wang et al., 2017), RRID: SCR_006786. Gene ontology (GO) terms with false discovery rate (FDR) < 10% were considered significantly enriched.

### Immunofluorescence Assay

Fibroblasts were plated on glass cover slips and cultured in macromolecularly crowded medium containing 37.5 mg/ml Ficoll PM70 (Sigma, F2878), 25 mg/ml Ficoll PM400 (Sigma, F4375) and 0.5% fetal bovine serum (Gibco, 10270-106) and replenished daily with 50 μg/ml of freshly prepared ascorbic acid (Fluka, 95210) to accelerate the deposition of extracellular matrix proteins as previously described (Kumar et al., 2015). After 4 days, samples were fixed in 4% formaldehyde for 15 mins at room temperature and washed three times with Phosphate-Buffered Saline (PBS). Samples were incubated for 1 h at room temperature with blocking buffer containing 1% bovine serum albumin (Sigma, A3912) in PBS and subsequently incubated overnight at 4°C with antibodies against collagen type IV (Abcam, ab86042, RRID: AB_1924897). The samples were then washed with PBS 3 times, incubated for 1 h at room temperature with Alexa Fluor 594-conjugated anti-mouse IgG antibodies (Invitrogen, A11020, RRID :AB_141974), washed with PBS 3 times and distilled water once before mounting on glass slides using Fluoroshield containing the nuclear stain 4′,6-diamidino-2-phenylindole (Sigma, F6057). Imaging was performed using a Leica DMi8 microscope and a Leica DFC3000 G camera integrated with a charge-coupled device sensor.

### Quantitative RT-PCR for Validation

Candidate DEGs that are either known target genes of S2P substrates and/or functionally involved in extracellular matrix, bone or cartilage metabolism were selected for validation by quantitative reverse transcription polymerase chain reaction (qRT-PCR) in four independent replicates. RNA was harvested using RNeasy Mini Kit (QIAGEN, 74104) according to manufacturer’s instructions and reverse transcribed to cDNA using the High-Capacity RNA-to-cDNA Kit (Applied Biosystems, 4387406). cDNA was diluted in RNase-free water to 3 ng/μl for qRT-PCR using TaqMan assays (Supplementary Table 2) on a 7900HT Fast Real-Time PCR System machine (Applied Biosystems). Fold change in gene expression was calculated by the 2^–ΔΔ*Ct*^ method – the gene expression levels were first normalized to the geometric mean of the cycle threshold (Ct) values of housekeeping genes *GAPDH*, *IPO8*, and *TBP* as an endogenous control within each sample; the fold change in gene expression in patient cells were then calculated against healthy controls.

### Lipid Analysis

Fibroblasts were passage into T75 (fatty acid analysis) or T150 (sterol analysis) flasks and grown in standard conditions described in section “Subjects and Cell Culture” and harvested 4 days after passaging by trypsinization. The cells were collected by centrifugation at 930 rpm for 5 mins at room temperature, washed once in PBS, and then pelleted by centrifugation at 8000 rpm for 5 min at 4°C. The PBS was removed and the cell pellets were stored at −80°C until ready for lipid analysis by mass spectrometry.

The determination of fatty acids in fibroblasts was carried out by gas chromatography coupled to a tandem mass spectrometer (GC-MS/MS, Thermo Scientific TSQ 8000, Waltham, MA, United States), according to the method of Moser et al. (1981). Briefly, the fatty acids in fibroblasts were extracted with a mixture of methanol and dichloromethane using a TissueLyser II according to the manufacturer’s instructions (Qiagen, Basel, Switzerland), and the organic phase was derivatized with acetyl chloride. The resulting fatty acid methyl esters were purified by liquid-liquid extraction with hexane. The samples were then injected into the GC-MS/MS system and recorded using selective reaction monitoring (Herter-Aeberli et al., 2019).

Sterol metabolites in cell homogenates were determined using a modified GC-MS method after liquid–liquid extraction (Kelley, 1995). n-hexane (LiChrosolv^®^) was obtained from Sigma-Aldrich Chemie GmbH (Taufkirchen, Germany). Stock solutions of standard sterols (Sigma-Aldrich Chemie GmbH, Taufkirchen, Germany) were prepared in ethanol (Carl Roth GmbH, Karlsruhe, Germany) at a concentration of 1 mg/ml. The internal standard (IS), 5α-Cholestane (Sigma-Aldrich Chemie GmbH, Taufkirchen, Germany), was prepared as a 0.1 mg/ml stock solution. For sample preparation, 160–200 μl of a cell suspension corresponding to around 0.5 mg protein was used. 100 μl of 0.25 mM 5α-Cholestane was added as internal standard to each sample. For saponification, samples were heated with 2 ml of 4% potassium hydroxide (Carl Roth GmbH, Karlsruhe, Germany) solution in ethanol at 60°C for 60 min in a heating block. The reaction mixture was diluted with 2.1 ml of purified water and acidified with 250 μl of 5N hydrochloric acid (HCl). After addition of solid sodium chloride the samples were extracted twice with 4 mL hexane. The combined hexane fractions were dried over anhydrous sodium sulfate and then the solvent was evaporated at 60°C under a stream of nitrogen. Samples were derivatized with 50 μl N-methyl-N-(trimethylsilyl)heptafluorobutyramide (MSHFBA, Macherey-Nagel, Düren, Germany) for 1 h at 60°C and diluted with 50 μl hexane prior to injection. For GC/MS analysis, the quadrupole mass spectrometer MSD 5977A (Agilent, Santa Rosa, CA, United States) was run in the selective ion-monitoring mode with electron impact ionization. Gas chromatographic separation was achieved on a capillary column (DB-5MS, 30 m × 0.25 mm; film thickness: 0.25; J&W Scientific, Folsom, CA, United States) using helium as carrier gas. A volume of 1 μL of the derivatized sample was injected in split mode. GC temperature parameters were 100°C for 2 mins, ramp 35°C/min to 300°C. Injector temperature was set to 280°C and interface temperature to 290°C. Quantification ions for sterols of interest were m/z 217 (5α-cholestane), m/z 329 (cholesterol), m/z 351 (7-dehydrocholesterol), m/z 372 (desmosterol), m/z 255 (lathosterol), m/z 393 (lanosterol), and m/z 349 (7-dehydrodesmosterol).

### Electron Microscopy Analysis

Fibroblasts from confluent T75 flasks were fixed with 3% glutaraldehyde in 0.1 M cacodylate buffer for 1 h at room temperature. The cells were then washed three times with 0.1 M cacodylate buffer for 5 mins per washing step to remove the fixation solution, harvested with a cell scraper, centrifuged at 13,000 rpm for 10 mins, washed three times with 0.1 M cacodylate buffer for 5 mins per washing step, post-fixed in 1% osmium tetroxide, dehydrated by a graded series of ethanol and embedded in Epon overnight at 65°C. Semi-thin and ultra-thin sections of embedded cells were prepared with an ultramicrotome and viewed with a transmission electron microscopy at the Center for Microscopy and Image Analysis, University of Zurich.

## Results

### Transcriptome Profiling and Differential Expression Analysis

**FIGURE 2:**
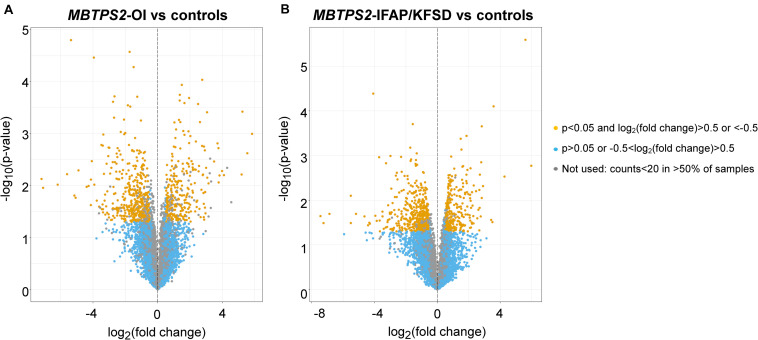
Volcano plots of transcriptome profiles of panels **(A)** *MBTPS2*-OI and **(B)** *MBTPS2*-IFAP/KFSD patient-derived fibroblasts compared to healthy control fibroblasts.

Volcano plots illustrating the statistical significance against fold change in gene expression between *MBTPS2*-OI and control or between *MBTPS2*-IFAP/KFSD and control cells cultured at basal conditions (no treatment) are depicted (Figure 2). In skin fibroblasts derived from *MBTPS2*-OI patients, RNA-sequencing found 841 DEGs in comparison to controls, of which 330 genes were upregulated and 511 were downregulated in the patients’ cells (Figure 2A). When fibroblasts derived from *MBTPS2*-IFAP/KFSD were compared to controls, 838 DEGs were identified, of which 321 were upregulated and 517 were downregulated in these patients’ skin fibroblasts (Figure 2B). 1540 DEGs were identified between *MBTPS2*-OI and *MBTPS2*-IFAP/KFSD, of which 888 were upregulated and 652 were downregulated in *MBTPS2*-OI compared to *MBTPS2*-IFAP/KFSD. The lists of DEGs in *MBTPS2*-OI and *MBTPS2*-IFAP/KFSD compared to controls can be found in Supplementary Tables 3, 4, respectively. Genes that are differentially expressed between *MBTPS2*-OI and *MBTPS2*-IFAP/KFSD are listed in Supplementary Table 5. 68 DEGs were upregulated in both *MBTPS2*-OI and *MBTPS2*-IFAP/KFSD compared to control whereas 119 DEGs were downregulated in both diseases (Supplementary Figure 1 and Supplementary Table 6). The observed numbers of overlapping DEGs are higher than what would be expected by chance based on the total number of genes identified by RNA-sequencing and the number of DEGs for each condition (Fisher’s exact test *p* < 0.0001).

A 5124 DEGs were identified in controls in response to thapsigargin treatment (2472 upregulated, 2652 downregulated), 4217 DEGs were identified in *MBTPS2*-OI in response to thapsigargin treatment (2191 upregulated, 2026 downregulated) and 5036 DEGs were identified in *MBTPS2*-IFAP/KFSD in response to thapsigargin treatment (2509 upregulated, 2527 downregulated). Furthermore, in response to thapsigargin treatment, 184 genes were upregulated and 213 genes were downregulated in *MBTPS2*-OI compared to controls, whereas 191 genes were upregulated and 171 genes were downregulated in *MBTPS2*-IFAP/KFSD compared to controls. 532 DEGs were observed between thapsigargin-treated *MBTPS2*-OI cells and *MBTPS2*-IFAP/KFSD cells, of which 247 were upregulated and 285 were downregulated in *MBTPS2*-OI.

The multivariate data analysis method principal components analysis (PCA) was performed to visualize the degree of similarities between gene expression profiles of the control and patient fibroblasts in a two-dimensional plot. Although no strong clustering of control and patient samples were observed, fibroblasts from patients with OI appear more similar to each other as did fibroblasts from IFAP/KFSD patients under basal conditions. Larger variations within the control samples are observed, where controls 1 and 4 were more similar to each other but different from controls 2 and 3. The strong effects of thapsigargin on gene expression changes are also observed, as seen by clustering of untreated samples and samples treated with thapsigargin (Supplementary Figure 2).

Pearson’s correlation coefficient was also analyzed for each pair of samples (Supplementary Figure 3). The strong effects of thapsigargin on gene expression changes are evident from the lower values of correlation coefficient obtained between no treatment (nt) and thapsigargin-treated (TG) samples. At basal conditions (nt), the IFAP and KFSD samples are more similar to each other (higher coefficient values as depicted by lighter shade of gray) than to OI samples (lower coefficient values as depicted by darker shade of gray). Likewise, OI samples are more similar to one another than to IFAP/KFSD samples. The larger variation in controls is also evident from the correlation coefficients, similar to the observations from the PCA analysis.

Next, a list of known target genes was generated through a search on the Harmonizome database (Rouillard et al., 2016) for TRANSFAC-curated transcription factor targets and a literature search for ATF6 (Jang et al., 2012; Shoulders et al., 2013; Chiang et al., 2017; Mügge and Silva, 2017; Dvela-Levitt et al., 2019), OASIS (Vellanki et al., 2010; Okuda et al., 2014), BBF2H7 (Izumi et al., 2012; Kondo et al., 2018), and SREBP (Horton et al., 2003; Sharpe and Brown, 2013). The isoforms of SREBP (SREBP1a, -1c encoded by *SREBF1* and SREBP2 encoded by *SREBF2*) were collectively analyzed since many of the target genes are transcriptionally regulated by both SREBP1 and SREBP2 (Horton et al., 2003). Heatmaps were generated to visualize the expression of these known target genes of ATF6, OASIS, BBF2H7, and SREBP in control, *MBTPS2*-OI and *MBTPS2*-IFAP/KFSD fibroblasts at basal conditions (no treatment, nt) or upon ER stress induction by thapsigargin (TG) treatment. The heatmaps are generated using the R-Function: heatmap.2 from package gplots, with the parameter Rowv = TRUE. For each gene, the colors indicate the log2 difference relative to the average of all samples. The order of the samples depicted in the heatmaps follow that listed in Supplementary Table 1.

**FIGURE 3:**
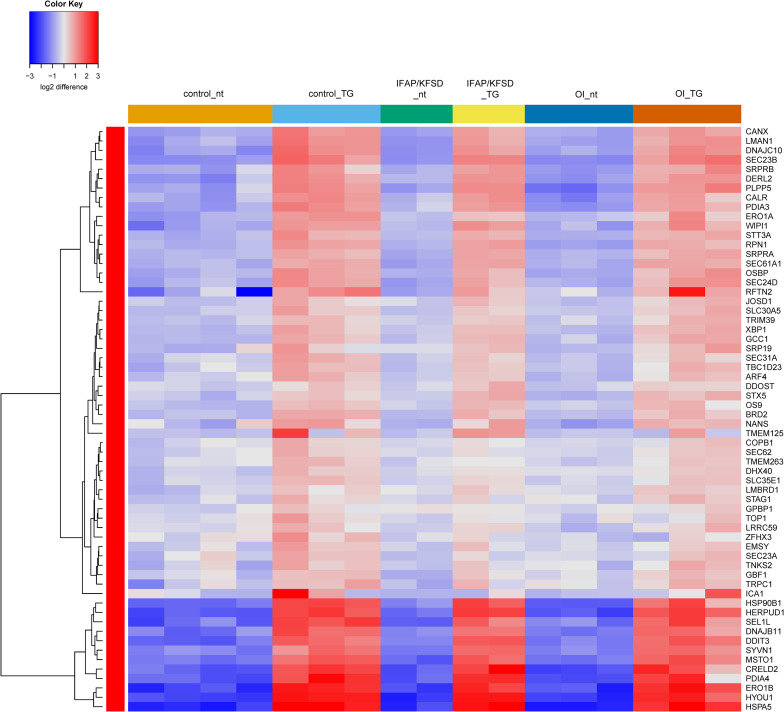
Heatmap depicting expression levels of genes that are transcriptionally regulated by ATF6 at basal conditions (nt: no treatment) and in the presence of thapsigargin (TG). The dendrogram on the left y-axis shows clustering of the genes.

**FIGURE 4:**
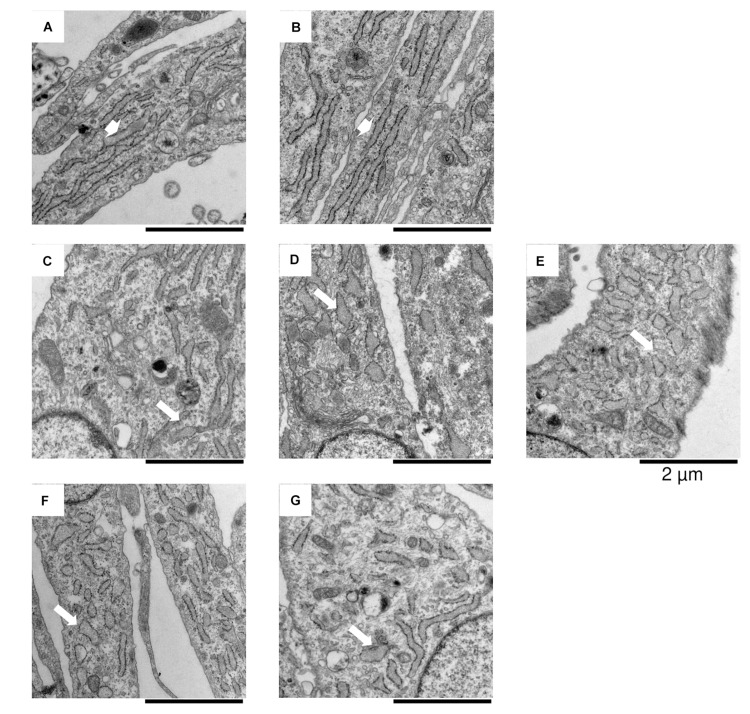
*In vitro* electron microscopy analyses of fibroblasts derived from **(A,B)** healthy controls, **(C–E)** *MBTPS2*-OI and **(F,G)** *MBTPS2-*IFAP/KFSD patients. In each image a portion of the fibroblasts is depicted with abundant ER cisterns. Short and large shaped ER cisterns are shown in the *MBTPS2*-OI [**(C–E)**, white arrows] and *MBTPS2-*IFAP/KFSD patients [**(F,G)**, white arrows] in contrast to the long and slender shaped ER cisterns shown in the two controls [**(A,B)**, white large arrows].

The induction of ATF6-dependent genes in response to thapsigargin is comparable in control, OI and IFAP/KFSD fibroblasts (Figure 3) suggesting that the ATF6-driven ER stress response is not altered by pathogenic variants in *MBTPS2*. ATF6-dependent genes are also not differentially expressed between control cells and patient cells at basal conditions (Supplementary Figure 4). This is in contrast with our previous observations by overexpression of wildtype and mutant MBTPS2 constructs with p5xATF6-luciferase reporter assay (Lindert et al., 2016). Nevertheless, electron microscopy analyses of fibroblast cultures *in vitro* revealed the presence of abnormally shortened and enlarged ER cisterns in *MBTPS2*-OI and *MBTPS2*-IFAP/KFSD patient fibroblasts in contrast to longer and slender ER cisterns present in control fibroblasts (Figure 4), suggesting that pathogenic variants of *MBTPS2* impose some form of cellular stress in both OI and IFAP/KFSD.

**FIGURE 5:**
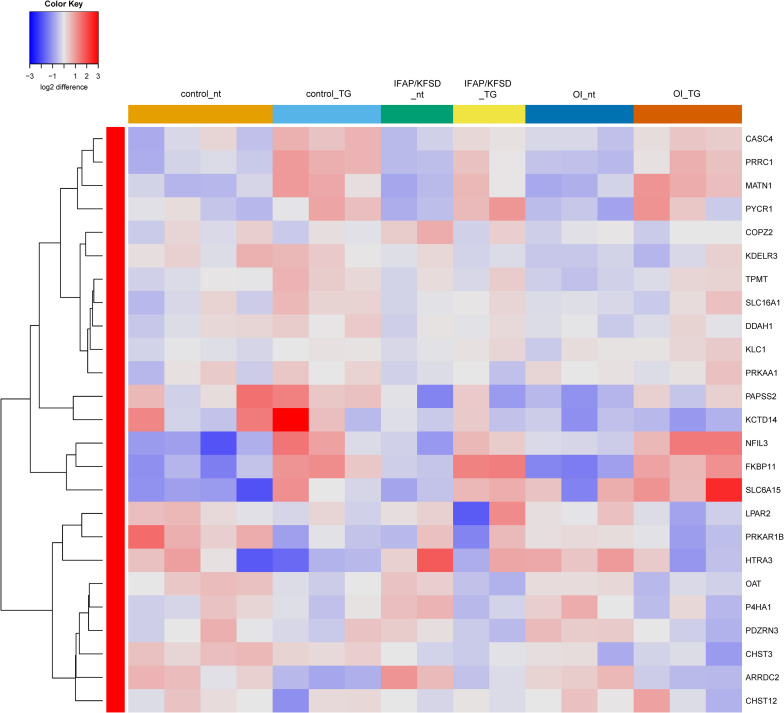
Heatmap depicting expression levels of genes that are transcriptionally regulated by OASIS at basal conditions (nt: no treatment) and in the presence of thapsigargin (TG). The dendrogram on the left y-axis shows clustering of the genes.

**FIGURE 6:**
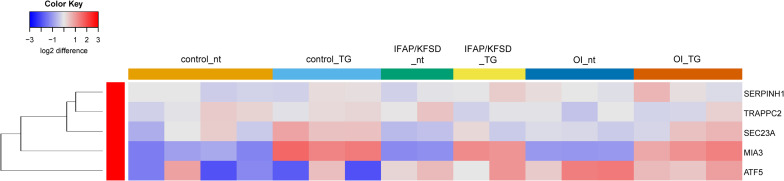
Heatmap depicting expression levels of genes that are transcriptionally regulated by BBF2H7 at basal conditions (nt: no treatment) and in the presence of thapsigargin (TG). The dendrogram on the left y-axis shows clustering of the genes.

Additionally, we previously observed defective cleavage of OASIS in *MBTPS2*-OI and *MBTPS2*-IFAP/KFSD patient fibroblasts compared to controls (Lindert et al., 2016). However, RNA-sequencing revealed downregulation of only *PAPSS2* and *CHST3* expression whilst the other known OASIS-dependent genes are not differentially expressed in *MBTPS2*-OI and *MBTPS2*-IFAP/KFSD fibroblasts compared to controls (Supplementary Figure 5). Unlike the strong induction of ATF6-dependent genes upon thapsigargin treatment, thapsigargin caused the upregulation of a few OASIS-dependent genes including *FKBP11*, *MTN1*, *PRRC1*, and *NFIL3*, with similar effects seen in patient and control cells (Figure 5). Furthermore, the expression of BBF2H7-dependent genes *SEC23A*, *SERPINH1*, *MIA3* and *TRAPPC2* is also not differentially expressed in both *MBTPS2*-OI and *MBTPS2*-IFAP/KFSD fibroblasts compared to controls, while the BBF2H7 target gene anti-apoptotic factor *ATF5* is upregulated in *MBTPS2*-OI and *MBTPS2*-IFAP/KFSD fibroblasts compared to controls (Supplementary Figure 6). Upregulation of *SEC23A* and *MIA3* in response to thapsigargin treatment was observed in patient and control cells (Figure 6). A plausible explanation for the minor differences observed in the expression of OASIS- and BBF2H7-dependent genes in S2P-defective cells is that skin fibroblasts are not physiologically relevant cell models for detecting alterations in OASIS and BBF2H7 activities, which play major roles in the development of osteoblasts in the bone and chondrocytes in the cartilage, respectively.

**FIGURE 7:**
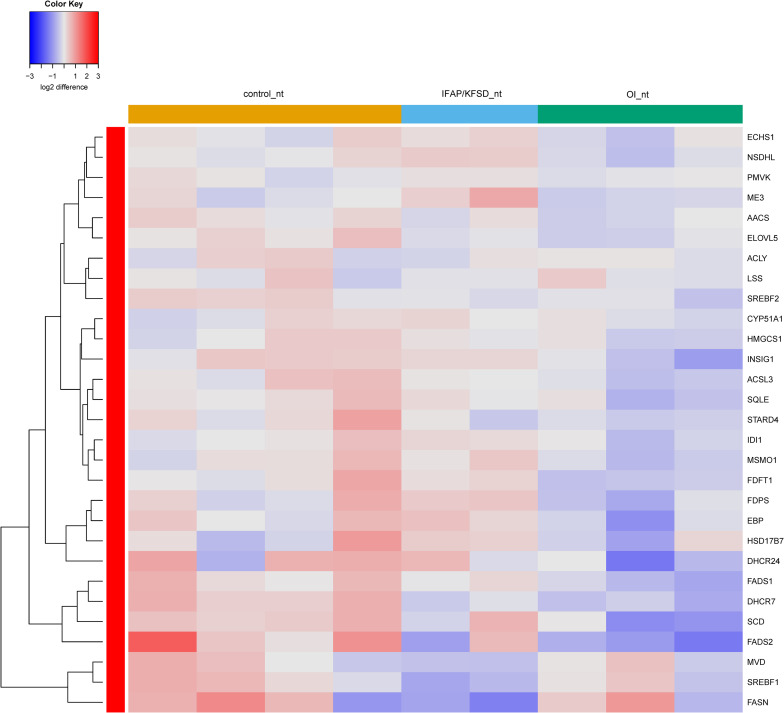
Heatmap depicting expression levels of SREBP-dependent genes at basal conditions. The dendrogram on the left y-axis shows clustering of the genes.

Interestingly, the expression of SREBP-dependent genes is suppressed in both *MBTPS2*-OI and *MBTPS2*-IFAP/KFSD patient fibroblasts compared to controls at basal condition. This effect was more pronounced in *MBTPS2*-OI than in *MBTPS2*-IFAP/KFSD patients’ cells (Figure 7). Thapsigargin treatment led to an induction of several SREBP-dependent genes in the fibroblasts except for cells derived from the *MBTPS2*-OI patient with the c.1376A > G (p.Asn459Ser) variant (Supplementary Figure 7). These data suggest that the activity of SREBP in activating transcription of its target genes is impaired in cells carrying pathogenic variants of *MBTPS2*, with a stronger impairment seen in cells with OI variants than in cells with IFAP/KFSD variants.

### Gene Ontology

Given the limited availability of patient-derived cells due to the rarity of the disease and the novelty of the described pathogenic variants in *MBTPS2*, the sample size in our study is inevitably too small to generate meaningful FDR for the differences in gene expression between patient and control cells by RNA-sequencing. Therefore, we used a more stringent *p*-Value cutoff of *p* < 0.01 (instead of *p* < 0.05) and log_2_ fold change > 0.5 or < −0.5 to generate a list of DEGs for ORA using the RNA-sequencing dataset. ORA was performed using the WebGestalt bioinformatics toolkit (Zhang et al., 2005; Wang et al., 2017) to identify biological processes, cellular components and molecular functions that are over-represented by the DEGs in *MBTPS2*-OI and in *MBTPS2*-IFAP/KFSD compared to healthy control fibroblasts.

**FIGURE 8:**
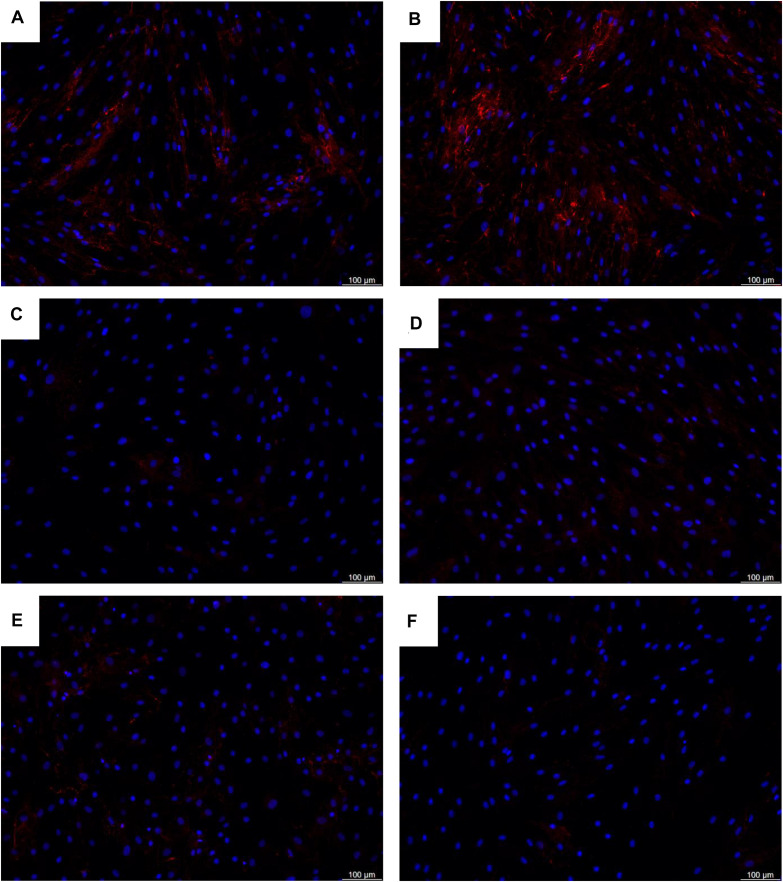
Deposition of collagen type IV proteins in the extracellular matrix by **(A,B)** healthy control fibroblasts, **(C,D)** *MBTPS2-*OI patient fibroblasts, and **(E,F)** *MBTPS2-*IFAP/KFSD patient fibroblasts was determined by immunofluorescence staining. Collagen type IV proteins are depicted in red and nuclei are depicted in blue.

ORA results are summarized in Table 1 for *MBTPS2*-OI and in Table 2 for *MBTPS2*-IFAP/KFSD. The analysis revealed that genes which code for extracellular matrix (ECM) proteins (GO term GO:0031012) were over-represented in both *MBTPS2*-OI and *MBTPS2*-IFAP/KFSD patient-derived fibroblasts in comparison to controls. Comparison of the DEGs sharing the GO term “ECM” in *MBTPS2*-OI and *MBTPS2-*IFAP/KFSD revealed that the genes *CCN5*, *DCN* and *TGFBR3* were upregulated in both *MBTPS2*-OI and *MBTPS2-*IFAP/KFSD compared to healthy controls whereas *COL4A1*, *COL4A2* and *GPC1* were downregulated. Furthermore, the downregulation in *COL4A1* and *COL4A2* gene expression translated into a reduction in deposition of collagen type IV protein in the extracellular matrix by both *MBTPS2*-OI and *MBTPS2-*IFAP/KFSD patient fibroblasts compared to controls (Figure 8).

**TABLE 1:** Over-represented GO terms amongst the DEGs in *MBTPS2*-OI vs. controls.

Gene ontology	Description	Enrichment ratio	*p*-Value	FDR	Gene set size	Overlap
***MBTPS2*-OI: Biological process**						
GO:0072376	protein activation cascade	9.31	3.74E-05	7.95E-03	35	6
GO:1990868	response to chemokine	9.31	3.74E-05	7.95E-03	35	6
GO:0006959	humoral immune response	9.17	1.21E-09	1.03E-06	77	13
GO:0031128	developmental induction	9.05	8.86E-04	4.03E-02	24	4
GO:0042303	molting cycle	5.14	4.07E-04	3.46E-02	74	7
GO:0007498	mesoderm development	4.81	6.07E-04	4.03E-02	79	7
GO:0001764	neuron migration	4.75	1.14E-04	1.38E-02	103	9
GO:0043588	skin development	4.58	2.16E-06	9.18E-04	166	14
GO:1990845	adaptive thermogenesis	3.95	9.40E-04	4.03E-02	110	8
GO:0030323	respiratory tube development	3.76	6.51E-04	4.03E-02	130	9
GO:0001763	morphogenesis of a branching structure	3.57	4.92E-04	3.80E-02	152	10
GO:0060326	cell chemotaxis	3.37	7.72E-04	4.03E-02	161	10
GO:0050900	leukocyte migration	3.18	1.31E-04	1.39E-02	239	14
GO:0001655	urogenital system development	3.16	8.18E-05	1.16E-02	258	15
GO:0060560	developmental growth involved in morphogenesis	3.16	1.28E-03	4.95E-02	172	10
GO:0008544	epidermis development	3.06	9.47E-04	4.03E-02	195	11
GO:0048638	regulation of developmental growth	3.06	3.38E-04	3.19E-02	231	13
GO:0019932	second-messenger-mediated signaling	2.91	8.82E-04	4.03E-02	224	12
GO:0002009	morphogenesis of an epithelium	2.77	5.41E-05	9.19E-03	372	19
GO:0031589	cell-substrate adhesion	2.75	9.23E-04	4.03E-02	257	13
GO:0060537	muscle tissue development	2.68	1.14E-03	4.62E-02	263	13
GO:0036230	granulocyte activation	2.45	8.50E-04	4.03E-02	355	16
***MBTPS2*-OI: Cellular Component**						
GO:0005581	collagen trimer	6.45	9.91E-04	4.00E-02	46	5
GO:0042581	specific granule	4.61	3.25E-04	2.80E-02	103	8
GO:0031012	extracellular matrix	3.84	1.81E-07	3.12E-05	309	20
GO:0043235	receptor complex	3.08	8.59E-04	4.00E-02	212	11
GO:0031253	cell projection membrane	2.97	1.16E-03	4.00E-02	220	11
***MBTPS2*-OI: Molecular Function**						
GO:0050840	extracellular matrix binding	7.82	1.04E-04	9.57E-03	43	6
GO:0001664	G protein-coupled receptor binding	4.84	6.43E-06	1.78E-03	139	12
GO:0048018	receptor ligand activity	3.87	2.93E-05	4.06E-03	188	13

**TABLE 2:** Over-represented GO terms amongst the DEGs in *MBTPS2*-IFAP/KFSD vs. controls.

Gene ontology	Description	Enrichment ratio	*p*-Value	FDR	Gene set size	Overlap
***MBTPS2-*IFAP/KFSD: Biological process**						
GO:0007422	peripheral nervous system development	8.54	6.75E-05	2.87E-02	56	6
GO:0007369	gastrulation	5.43	4.03E-05	2.87E-02	132	9
***MBTPS2-*IFAP/KFSD: Cellular Component**						
GO:0005796	Golgi lumen	10.24	2.29E-05	1.97E-03	50	6
GO:0031012	extracellular matrix	4.31	6.36E-07	1.09E-04	317	16
***MBTPS2-*IFAP/KFSD: Molecular Function**						
GO:0050840	extracellular matrix binding	9.39	1.76E-04	4.89E-02	43	5

Amongst the enriched GO terms that are listed in Tables 1, 2, several more might be potentially interesting in the understanding of the pathomechanisms and how the reviewed *MBTPS2*-mutations lead to two distinct phenotypes. These include skin development (GO:0043588), epidermis development (GO:0008544), cell-substrate adhesion (GO:0031589), collagen trimer (GO:0005581), receptor complex (GO:0043235) and ECM-binding (GO:0050840).

### qRT-PCR

**FIGURE 9:**
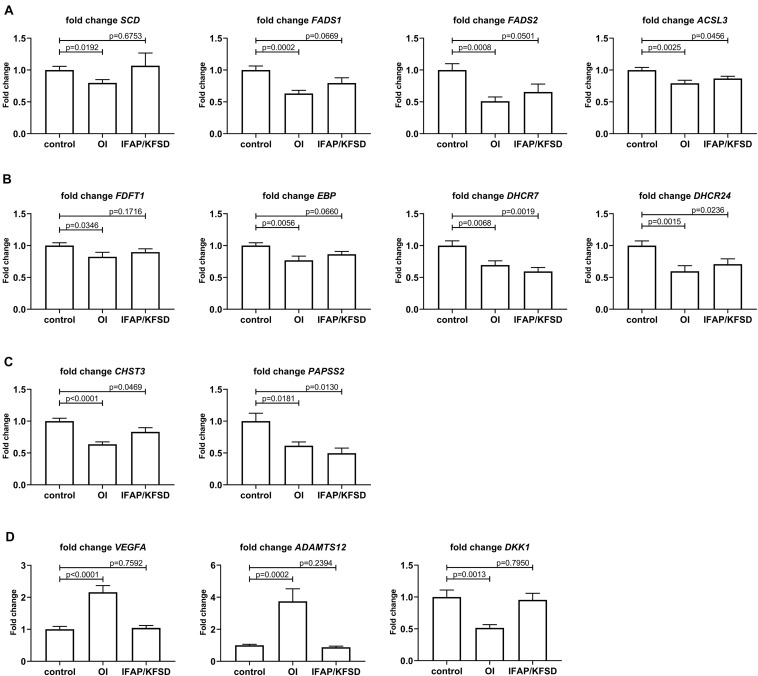
qRT-PCR was performed to validate the RNA-sequencing results for **(A)** SREBP-dependent genes involved in fatty acid metabolism, **(B)** SREBP-dependent genes involved in sterol metabolism, **(C)** OASIS-dependent genes involved in skeletal development, and **(D)** genes involved in bone or cartilage homeostasis that are differentially expressed in *MBTPS2*-OI compared to control. Gene expression was measured in four independent replicates per subject, and t-tests were performed for statistical analysis. Data are expressed as mean ± SEM.

Based on the above analyses suggesting impaired SREBP activity in *MBTPS2*-mutant cells, we selected several SREBP-dependent genes involved in fatty acid metabolism (*SCD*, *FADS1*, *FADS2*, and *ACSL3*) and sterol metabolism (*FDFT1*, *EBP*, *DHCR7*, and *DHCR24*) for validation by qRT-PCR; OASIS-dependent genes involved in skeletal development (*CHST3* and *PAPSS2*) were also selected. Furthermore, in the top hits of DEGs in *MBTPS2*-OI compared to controls we identified several genes involved in processes concerning either bone or cartilage homeostasis, which could contribute to the bone phenotype observed in OI patients. These genes, namely *VEGFA*, *ADAMTS12* and *DKK1* were also selected for validation by qRT-PCR (Figure 9).

By qRT-PCR, we observed a significant downregulation in the expression of *SCD*, *FADS1*, *FADS2* and *ACSL3* in *MBTPS2*-OI patient fibroblasts, and a trend towards suppression of *FADS1* and *FADS2* expression in *MBTPS2*-IFAP/KFSD patient fibroblasts (Figure 9A), in line with the RNA-sequencing data. A suppression of *ACSL3* expression was also observed in *MBTPS2*-IFAP/KFSD but to a smaller magnitude to that seen in *MBTPS2*-OI (Figure 9A), although this suppression was not significant by RNA-sequencing. Since these genes are involved in the synthesis of fatty acid, the data suggests perturbations in fatty acid metabolism in response to pathogenic *MBTPS2* variants, likely with a stronger effect in *MBTPS2*-OI than in *MBTPS2*-IFAP/KFSD. Therefore, fatty acid content was subsequently measured in cultured fibroblasts for functional validation of the transcriptomics results (see section “Fatty Acid Profile of Cells”).

In line with the RNA-sequencing data, the downregulation in the expression of *DHCR7* in both *MBTPS2*-OI and *MBTPS2*-IFAP/KFSD was confirmed by qRT-PCR; suppression of *FDFT1*, *EBP* and *DHCR24* in *MBTPS2*-OI compared to controls was also demonstrated (Figure 9B). In contrary to the RNA-sequencing data which showed only modest, non-significant suppression of *DHCR24* in *MBTPS2*-IFAP/KFSD, qRT-PCR revealed a significant downregulation of *DHCR24* in *MBTPS2*-IFAP/KFSD fibroblasts, albeit at a smaller fold change than that seen in *MBTPS2*-OI cells. Collectively, the dysregulation of SREBP-dependent genes involved in the cholesterol synthesis pathways point towards perturbed sterol metabolism caused by pathogenic mutations in *MBTPS2*. Thus, the sterol profile of these fibroblasts was subsequently determined (see section “Sterol Profile of Cells”).

The downregulation of *CHST3* in both *MBTPS2*-OI and *MBTPS2*-IFAP/KFSD and of *PAPSS2* in *MBTPS2*-OI was confirmed by qRT-PCR (Figure 9C). A significant suppression of *PAPSS2* expression was also observed in *MBTPS2*-IFAP/KFSD by qRT-PCR (Figure 9C), although this trend was not statistically significant (*p* = 0.0677) by RNA-sequencing.

Finally, the strong upregulation of *VEGFA* and *ADAMTS12* and downregulation of *DKK1* in *MBTPS2*-OI compared to both control and *MBTPS2-*IFAP/KFSD observed by RNA-sequencing was reproduced by qRT-PCR (Figure 9D).

### Fatty Acid Profile of Cells

**FIGURE 10:**
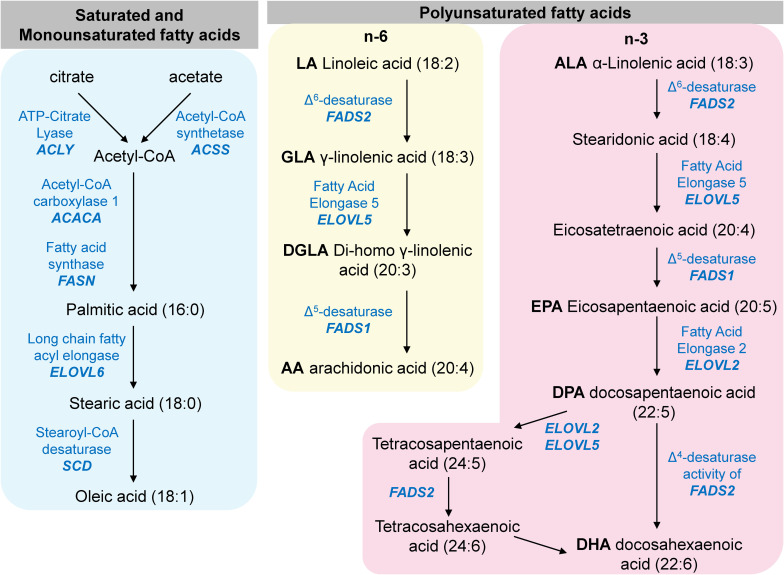
A simplified diagram of mammalian fatty acid metabolism. The enzymes listed in this diagram are encoded by genes that are transcriptionally regulated by SREBP.

The biosynthesis of non-essential fatty acids is catalyzed in sequential steps by many enzymes whose expression is regulated by SREBP at the transcript level, as depicted in a simplified diagram in Figure 10. Therefore, we quantified the cellular content of several of these fatty acids in control and patient fibroblasts by GC-MS/MS for the functional validation of the suppressed expression of SREBP target genes involved in fatty acid metabolism.

**FIGURE 11:**
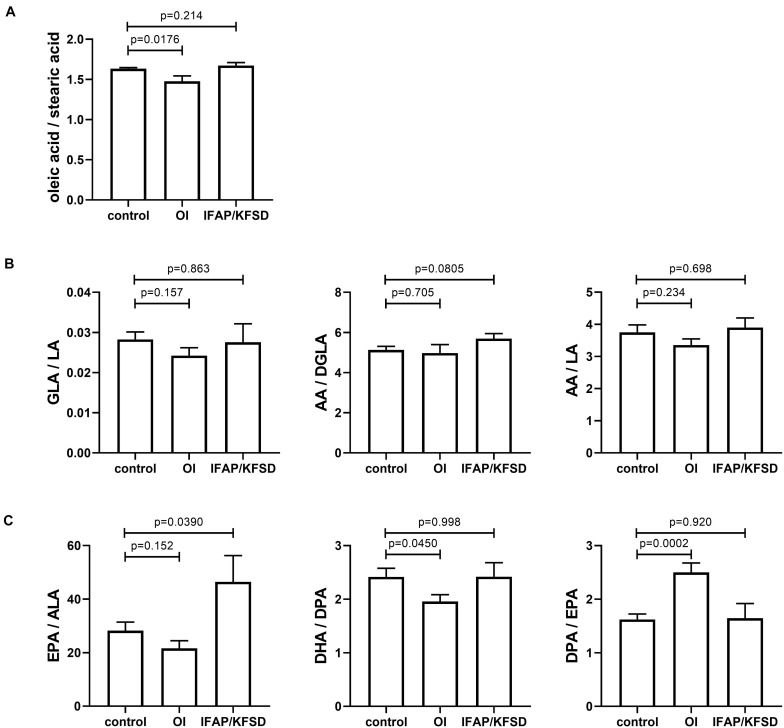
**(A−C)** The cellular content of several saturated, monounsaturated and polyunsaturated omega-6 and omega-3 fatty acids were determined by GC-MS/MS and expressed as ratios. Four independent measurements were performed per subject, and *t*-tests were performed for statistical analysis. Data are expressed as mean ± SEM.

The ratio of oleic acid to stearic acid is decreased in *MBTPS2*-OI fibroblasts compared to controls (Figure 11A), which demonstrates reduced stearoyl-CoA desaturase activity that converts stearic acid to oleic acid. This is consistent with the suppressed gene expression of *SCD* encoding stearoyl-CoA desaturase in *MBTPS2*-OI patient fibroblasts but not *MBTPS2*-IFAP patient fibroblasts (Figure 9A).

*FADS1* and *FADS2* encode the Δ^5^-desaturase and Δ^6^-desaturase enzymes, respectively, and are involved in the biosynthesis of long chain omega-6 fatty acids from linoleic acid and long chain omega-3 fatty acids from α-linolenic acid (Figure 10). In particular, the Δ^6^-desaturase enzyme is the rate-limiting enzyme in the metabolism of linoleic acid to arachidonic acid and of α-linolenic acid to docosapentaenoic acid (Horrobin et al., 1993; Cho et al., 1999). Since the gene expression of *FADS1* and *FADS2* is suppressed in *MBTPS2*-OI fibroblasts compared to controls (Figure 9A), we hypothesized alterations in the metabolism of omega-3 and omega-6 fatty acids in these cells.

For omega-6 fatty acids, the ratios of γ-linolenic acid to linoleic acid (GLA / LA), arachidonic acid to di-homo-γ-linolenic acid (AA / DGLA), and arachidonic acid to linoleic acid (AA / LA) are not significantly altered (Figure 11B) despite *FADS2* and *FADS1* suppression.

For omega-3 fatty acids, the ratio of eicosapentaenoic acid to α-linolenic acid (EPA / ALA) is not significantly altered in *MBTPS2*-OI fibroblasts (Figure 11C). However, in *MBTPS2*-OI fibroblasts, a significant reduction in the ratio of docosahexaenoic acid to docosapentaenoic acid (DHA / DPA) is observed (Figure 11C), consistent with the suppression of *FADS2*, which can convert DPA to DHA directly through its Δ^4^-desaturase activity or via tetracosapentaenoic acid and tetracosahexaenoic acid with its Δ^6^-desaturase activity. On the other hand, the ratio of docosapentaenoic acid to eicosapentaenoic acid (DPA / EPA) is significantly increased in *MBTPS2*-OI fibroblasts (Figure 11C). This elevated ratio is not surprising since the reduction in *FADS2* and *FADS1* expressions can block the synthesis of EPA from ALA and at the same time lead to an accumulation in DPA due to a block in the desaturation of DPA to DHA.

### Sterol Profile of Cells

The biosynthesis of cholesterol is a multi-step process catalyzed by several enzymes whose expression is transcriptionally regulated by SREBP. The first few steps require the conversion of acetyl-CoA to 3-hydroxy- 3-methylglutaryl-CoA (HMG-CoA) and then to mevalonate. Mevalonate undergoes phosphorylation, decarboxylation and condensation to form lanosterol, which is the first sterol synthesized in this pathway. Subsequently, the conversion of lanosterol to cholesterol occurs via the Bloch pathway (involving the sterol intermediates 7-dehydrodesmosterol and desmosterol) or the Kandutsch-Russell pathway (involving the sterol intermediates lathosterol and 7-dehydrocholesterol), or a hybrid of both pathways, catalyzed by multiple enzymes including *EBP*, *DHCR7* and *DHCR24* (Kandutsch and Russell, 1960; Bloch, 1965; Mitsche et al., 2015). Since RNA-sequencing and qRT-PCR demonstrated the downregulation of *EBP*, *DHCR7* and *DHCR24* in *MBTPS2-*mutant cells, we attempted to quantify the cellular content of the sterol metabolites in control and patient fibroblasts. In consideration of previous findings in which cells lacking endogenous *Mbtps2* do not survive in cholesterol-deficient conditions and overexpression of wildtype but not mutant *MBTPS2* rescues this phenotype (Oeffner et al., 2009), we cultured our fibroblasts in standard sterol-replete medium. Intracellular levels of lanosterol, 7-dehydrodesmosterol, desmosterol, lathosterol, 7-dehydrocholesterol and cholesterol were measured by GC-MS/MS; ratios of these sterol metabolites were calculated to assess the conversion of one metabolite to another. Unfortunately, we could not detect consistent alterations in the sterol profiles of the control and patient fibroblasts (Supplementary Figure 8), likely due to the presence of sterols in the culture medium that can cause feedback inhibition of HMG-CoA reductase thus resulting in a reduction in endogenous cholesterol biosynthesis. Therefore, the effects of *MBTPS2* mutations in OI and IFAP/KFSD on sterol metabolism and cholesterol synthesis remains to be better characterized.

## Discussion

*MBTPS2* mutations have been identified as the first X-linked cause of OI that is associated with a moderate to severe, potentially intrauterine lethal clinical phenotype (Lindert et al., 2016). Peculiarly, *MBTPS2* pathogenic variants distinct from those identified in OI were already known to cause the dermatological spectrum condition IFAP and KFSD (Oeffner et al., 2009; Aten et al., 2010; Bornholdt et al., 2013). This offers a unique biological context to identify molecular players and mechanisms that are specifically perturbed in *MBTPS2*-OI but not in *MBTPS2*-IFAP/KFSD and are thus relevant to the pathophysiology in the skeletal system.

Through transcriptome profiling of fibroblasts obtained from OI patients, IFAP/KFSD patients and healthy donors, we identified unique gene expression changes in OI cells; these genes play biological roles in bone and cartilage development, or regulate lipid metabolism. Furthermore, we found that the relative abundance of fatty acids in OI fibroblasts were altered. These *in vitro* findings generate new insights on biological molecules and pathways that could be relevant to the progression of OI and open up new hypotheses on the pathomechanisms underlying OI that should be further tested in more relevant *in vitro* and *in vivo* models of bone and cartilage. Importantly, this will pave the way towards the development of OI-centric therapeutic strategies.

In the remaining discussion section of the manuscript, we highlight the biological functions of genes that are differentially expressed in *MBTPS2-*OI and their potential contribution to the pathogenesis of OI.

### SREBP-Dependent Genes

*EBP* (Emopamil-binding protein, also known as EBP cholestenol delta-isomerase) is significantly downregulated in *MBTPS2*-OI patient fibroblasts (Figure 9B and Supplementary Table 3) and displays a trend towards downregulation in *MBTPS2*-IFAP/KFSD patient fibroblasts (*p* = 0.0660, Figure 9B). EBP cholestenol delta-isomerase catalyzes the conversion of Δ^8^-sterols to their corresponding Δ^7^-isomers in cholesterol biosynthesis. Loss-of-function mutations in *EBP* on the X-chromosome have been previously identified in Conradi-Hünermann-Happle-syndrome, which is also known as X-linked dominant chondrodysplasia punctata 2 (CDPX2) (OMIM #302960). Clinical manifestations of CDPX2 include short stature, skeletal abnormalities consisting of rhizomelic shortening of the limbs, stippling and scoliosis, skin abnormalities including scaling ichthyosis and coarse scalp hair with scarring alopecia (Kumble and Savarirayan, 2020). Strikingly, these clinical features appear to partially overlap with that of *MBTPS2*-OI patients in terms of short stature and skeletal abnormalities, as well as with that of IFAP and KFSD patients with skin abnormalities. Therefore, deficiency in EBP cholestenol delta-isomerase affecting sterol metabolism seems to affect multiple tissues.

*ACSL3* encodes the Long-chain-fatty-acid-CoA ligase 3 which catalyzes the production of long chain fatty acyl-CoA esters from free long-chain fatty acids, which then serve as a substrate for lipid synthesis and β-oxidation. Thus, a downregulation of *ACSL3* observed in *MBTPS2* mutant cells (Figure 9A) may alter cellular metabolism, energy production and cell survival, similar to that observed in lung cancer cells (Padanad et al., 2016). The involvement of ACSL3 in inducing osteoblastic differentiation of vascular smooth muscle cells has also been demonstrated as one of the pathophysiological processes in the development of vascular calcification. Inhibition of ACSL3 blocked palmitic acid-induced osteoblastic differentiation of vascular smooth muscle cells as evident from attenuated expression of *BMP2* and *MSX2* (Kageyama et al., 2013). Furthermore, in a study performed in a cohort of 91 females, *ACSL3* was found to be correlated to bone mineral density (Reppe et al., 2010). These suggest that ACSL3 plays a role in maintaining bone health and development, although the mechanism(s) remains to be elucidated.

Our data also demonstrated reduced expression of several SREBP-dependent genes involved in fatty acid metabolism and changes in fatty acid relative abundance in *MBTPS2-*OI cells. This is an interesting finding since numerous studies have shown an association between fatty acid and bone health (reviewed by Salari et al., 2008; Mangano et al., 2013; Wauquier et al., 2015). A high dietary intake of the monounsaturated fatty acid oleic acid positively regulates calcium absorption, bone mineralization and bone density in mice (Rezq et al., 2010) and prevents ovariectomy-induced osteoporosis in rats (Saleh and Saleh, 2011). In humans, a positive association between high oleic acid intake through the consumption of olive oil and bone mineral density has also been observed (Trichopoulou et al., 1997; Roncero-Martín et al., 2018). For polyunsaturated fatty acids (PUFA), cross-sectional and longitudinal studies in various populations have led to variable observations (Mangano et al., 2013). However, more recent randomized control trials suggest improved bone status with long-chain PUFA supplementation (Martin-Bautista et al., 2010; Lappe et al., 2013; Mangano et al., 2013). A 6-month intervention with genistein, vitamin D3, vitamin K1 and PUFA (EPA and DHA) improved the bone mineral density in post-menopausal women (Lappe et al., 2013). In another study, a 12-month intervention with EPA, DHA, oleic acid, and several vitamins including vitamin D led to an improvement in the patients serum of the bone formation biomarkers osteoprotegerin (OPG), receptor activator of nuclear factor κB ligand (RANKL), OPG/ RANKL ratio and osteocalcin (Martin-Bautista et al., 2010). However, in these studies, the multiple component nature of the interventions hinders a sound conclusion on the specific effects of long chain PUFA on bone health. In animal studies, EPA supplementation prevents the loss of bone weight and strength caused by estrogen deficiency or calcium deficiency (Sakaguchi et al., 1994) while a diet rich in DHA lead to higher bone mineral density and bone mineral content (Lukas et al., 2011) in rats. Long term dietary intake of EPA also improved the structural and mechanical properties of cortical bone in mice (Bonnet and Ferrari, 2011), while dietary long chain omega-3 PUFAs were protective against age-induced bone loss in rats (Shen et al., 2006). The mechanisms underlying the effects of these fatty acids on bone health has also been investigated (reviewed in Wauquier et al., 2015). For instance, omega-3 PUFAs can promote osteoblast differentiation via increased production of insulin-like growth factor-1 and parathyroid hormone (Shen et al., 2006). Furthermore, lipids are required to form vesicles for transporting vitamins D and K, which are involved in regulating serum calcium and phosphorous concentrations and activation of osteocalcin for bone mineralization (Tintut and Demer, 2014). Therefore, the significant suppression of *SCD*, *FADS1* and *FADS2* specifically in *MBTPS2*-OI patient fibroblasts and the resultant hindered conversion of stearic acid to oleic acid, ALA to EPA and DPA to DHA may negatively affect bone metabolism and health. In a recent study (Wang et al., 2020), monoallelic pathogenic variants in *SREBF1* encoding for the SREBP1 transcription factor were found to cause IFAP in individuals who do not carry pathogenic variants of *MBTPS2*. The *SREBF1* mutations lead to impaired cleavage of SREBP1 by S1P, thereby hindering the translocation of SREBP1 to the nucleus. RNA-sequencing of skin from the scalp of affected individuals revealed the downregulation of genes involved in lipid metabolism, although the serum lipid profiles of the patients appeared normal. Of particular interest to us, the downregulation of *SCD* in the *SREBF1*-IFAP patients match our findings of downregulation of *SCD* in the *MBTPS2-*OI fibroblasts. However, *SCD* was not downregulated in *MBTPS2*-IFAP/KFSD fibroblasts. Despite significant downregulation of *SCD* with consequent alterations in the relative abundance of fatty acids in *MBTPS2*-OI fibroblasts, the *MBTPS2*-OI patients in our study did not present with alopecia or photophobia.

### OASIS-Dependent Genes

In the list of OASIS-dependent genes, *CHST3* and *PAPSS2* were significantly downregulated in both *MBTPS2*-OI and *MBTPS2*-IFAP/KFSD.

*CHST3* encodes chondroitin 6-O-sulfotransferase (C6ST-1), an enzyme that catalyzes the sulfation of chondroitin. Loss-of-function mutations in *CHST3* are causative of spondyloepiphyseal dysplasia (SED) Omani type (OMIM 143095) (Thiele et al., 2004), in which a missense mutation in *CHST3* leads to a generalized defect in the chain sulfation of chondroitin sulfate resulting in severe chondrodysplasia. Affected patients display normal length at birth but severely reduced height at adulthood, progressive kyphoscoliosis and joint dislocations leading to restricted joint movement (Thiele et al., 2004). Similar to the SED Omani type patients, *MBTPS2*-OI patients also have a short stature and both patients from the German pedigree presented with kyphoscoliosis.

*PAPSS2* encodes 3′-phosphoadenosie 5′-phosphosulfate (PAPS) synthase 2 which synthesizes PAPS, a universal donor of sulfate required for the sulfation of many glycosaminoglycans, proteins and steroid hormones. Specifically, PAPS is required for the sulfation of the glycosaminoglycan chondroitin sulfate, which is an important component of cartilage. Loss-of-function mutations in *PAPSS2* are associated with autosomal-recessive forms of brachyolmia, a heterogeneous group of skeletal dysplasias, including the Hobaek (OMIM 271530) and Toledo (OMIM 271630) types (Miyake et al., 2012; Iida et al., 2013). Affected individuals show short-trunk short stature, scoliosis and broad proximal interphalangeal joints. In our study, *PAPSS2* was significantly downregulated in the fibroblasts of both *MBTPS2*-OI and *MBTPS2*-IFAP/KFSD patients (Figure 9C), which might contribute to the two phenotypes in a yet unknown way.

### BBF2H7-Dependent Genes

Regulated intramembrane proteolysis involving S1P and S2P encoded by *MBTPS1* and *MBTPS2*, respectively, has been previously shown to be an important process in chondrogenesis. This is mediated by RIP activation of the BBF2H7 transcription factor, which in turn upregulates the expression of *SEC23A* (Saito et al., 2009), *SERPINH1*, *MIA3* and *TRAPPC2* (Kondo et al., 2018) essential for the formation of mega vesicles for ECM protein secretion in cartilage development. Schlombs et al. (2003) observed that knockdown of either *Mbtps1* or *Mbtps2* leads to cartilage defects in zebrafish as demonstrated by reduced Alcian blue staining. S1P deficiency caused by a mutation in *MBTPS1* was recently described in a pediatric patient with retarded growth and skeletal dysplasia presenting with reduced bone mineral density, spondyloepiphyseal dysplasia, kyphosis and dysmorphic facial features (Kondo et al., 2018). In this patient, the expression of BBF2H7-dependent genes *SEC23A*, *SERPINH1*, *MIA3*, and *TRAPPC2* crucial for ECM protein secretion was reduced, coupled with abnormal intracellular retention of collagen and enlarged ER (Kondo et al., 2018). Similarly, deletion of *Bbf2h7* in mice led to severe chondrodysplasia, abnormal expansion of the ER containing aggregated type II collagen and cartilage oligomeric matrix protein (COMP) and death by suffocation shortly after birth due to an immature chest cavity (Saito et al., 2009). Such defective chest cavity formation was also observed in our cohort of *MBTPS2*-OI patients in the form of pectus excavatum and pectus carinatum (Lindert et al., 2016). Abnormal, rounded ER morphologies were also observed in *MBTPS2*-OI and *MBTPS2*-IFAP/KFSD patient fibroblasts *in vitro* by electron microscopy analyses (Figure 4), although our transcriptomics analysis of patient-derived fibroblasts revealed only subtle differences in the expression of BBF2H7-dependent genes (Figure 5). In addition, an alternative mechanism of BBF2H7-regulated chondrogenesis has been proposed, in which the C-terminus fragment of BBF2H7 is secreted into the extracellular space upon RIP cleavage and binds directly to Indian hedgehog and its receptor Patched-1 to activate Hedgehog signaling and promote proliferation of chondrocytes (Saito et al., 2014). Therefore, it would be interesting to further investigate whether the pathogenic *MBTPS2* variants associated with either OI or IFAP/KFSD have different effects on BBF2H7 transcriptional activities and Hedgehog signaling causing impaired chondrogenesis in *in vitro* and *ex vivo* models of cartilage.

### Other Genes and Pathways Involved in Skeletal Development

Untargeted transcriptome analysis has delivered further evidence pointing towards perturbed skeletal development in *MBTPS2*-OI. Specifically, Vascular Endothelial Growth Factor A (*VEGFA*) and A Disintegrin And Metalloproteinase with Thrombospondin type 1 motif 12 (*ADAMTS12*) are strongly upregulated in *MBTPS2-*OI fibroblasts (Figure 9D and Supplementary Table 3).

Widely known for its role in angiogenesis, VEGFA (also known as VEGF before the discovery of other VEGF family members) is also involved in regulating growth plate morphogenesis, cartilage remodeling and bone formation (Gerber et al., 1999). The upregulation of *VEGFA* seen specifically in *MBTPS2*-OI could thus be associated with disturbed chondrocyte and bone development.

*ADAMTS12* encodes a zinc metalloproteinase which degrades ECM components. It is strongly expressed in growth plate cartilage and has a potential inhibitory effect on chondrocyte differentiation, highlighting its involvement in chondrogenesis (Bai et al., 2009). Pathologically, the upregulation of ADAMTS12 expression and cleavage of COMP by ADAMTS12 has been demonstrated in arthritis (Luan et al., 2008; Liu, 2009). ADAMTS12 is also overexpressed in endplate cartilage of patients with degenerative disc changes, coupled with a downregulation of several marker genes for chondrogenesis (Zhang et al., 2012b). Thus, its upregulation in *MBTPS2*-OI patient cells suggest its potential pathogenic involvement in skeletal development.

Hence, the determination of VEGFA and ADAMTS12 levels particularly in chondrocytes of *MBTPS2*-OI and *MBTPS2-*IFAP/KFSD patients would be highly valuable for understanding their involvement in the pathomechanisms related to the skeletal phenotype seen in OI. However, the mechanisms that contribute to elevated expression of *VEGFA* and *ADAMTS12* as a consequence of *MBTPS2* mutations in OI but not in IFAP/KFSD remains to be deciphered.

*DKK1* encodes the Dickkopf-1 protein, a secreted protein that antagonizes the Wnt/β-catenin signaling by binding and inactivating the lipoprotein-related protein LRP5/6 coreceptor. *DKK1* is transcriptionally regulated by several factors, including the Msx2 transcription factor highly expressed in osteoblasts, the BMP/c-Jun signaling pathway and via a negative feedback loop involving TCF/β-catenin (Grotewold and Rüther, 2002; Niida et al., 2004; Chamorro et al., 2005; Pinzone et al., 2009). DKK1 plays an important role in development; it promotes embryonic head induction and programmed cell death in the developing limb (Mukhopadhyay et al., 2001; Grotewold and Rüther, 2002) and also switches the mesenchymal stem cell differentiation pathway to favor adipogenesis over osteoblastogenesis and chondrogenesis thereby preventing endochondral bone formation (Christodoulides et al., 2006; Chen et al., 2007; Pinzone et al., 2009). In addition to inhibiting osteoblast differentiation and bone formation, elevated levels of DKK1 also promote bone resorption by inducing osteoclastogenesis via increased receptor activator of NF-kB ligand (RANKL) and decreased osteoprotegerin (OPG) production (Pinzone et al., 2009). Our transcriptomics analysis revealed a downregulation in the expression of *DKK1* in *MBTPS2-*OI patient fibroblasts (Figure 9D) suggesting that bone homeostasis may be perturbed, although its exact involvement and role in the pathomechanisms of OI requires more in-depth studies. In a recent transcriptomics study performed on whole blood of OI patients with *COL1A1* mutations (Zhytnik et al., 2020), the authors observed a decreased expression of *DKK3* from the same Dickkopf family of Wnt signaling antagonisers in *COL1A1*-OI patients compared to healthy controls. Therefore, further investigations in whether a reduction in Wnt signaling inhibition represents a common compensatory mechanism to avoid an impairment of bone formation in OI would be very interesting.

### Genes Encoding ECM Proteins

Genes encoding extracellular matrix (ECM) proteins (GO term GO:0031012) were found to be over-represented by ORA analysis of differentially expressed genes. *COL4A1*, *COL4A2*, and *GPC1* were downregulated whereas *CCN5*, *DCN*, and *TGFBR3* were upregulated in both *MBTPS2*-OI and *MBTPS2*-IFAP/KFSD compared to healthy controls.

#### Downregulated Genes *COL4A1, COL4A2*, and *GPC1*

The α1α1α2 heterotrimers of type IV collagen encoded by *COL4A1* and *COL4A2* constitute one of the most abundant components of nearly all basement membranes. After assembly, extensive post-translational modification and secretion into the ECM, they polymerize into a network and interact with other extracellular and membrane bound molecules. Mutations in *COL4A1* or *COL4A2* are pleiotropic and contribute to a spectrum of multi-system disorders including cerebrovascular, ocular, cerebral, renal and muscular defects (Kuo et al., 2012). *COL4A1* and *COL4A2* expression is downregulated by a family of microRNAs. In particular, miR-214-5p has been shown to suppress bone formation, and its inhibition in mouse osteoblastic cells MC3T3-E1 increases *COL4A1* and *COL1A1* expression and leads to increased cell viability. Similarly, the overexpression of *COL4A1* by plasmid transfection increases cell viability and *COL1A1* expression (Li et al., 2017). In line with this, we previously observed a reduction in the secretion rate of collagen type I protein by *MBTPS2-*OI and *MBTPS2-*IFAP/KFSD fibroblasts (Lindert et al., 2016). Therefore, the downregulation of *COL4A1* and *COL4A2* might negatively impact cell viability, osteoblast differentiation and general extracellular matrix properties of the bone in *MBTPS2*-OI.

*GPC1* encodes glypican-1, a cell surface anchored heparan sulfate proteoglycan. Its heparan sulfate side chain mediates binding to cytokines and growth factors thus facilitating the initiation and perpetuation of cell signaling (Matsuo and Kimura-Yoshida, 2013; Wang et al., 2019). Glypican-1 has been shown to promote the interaction between fibroblast growth factor 2 (FGF2) and its receptor FGFR1, thereby stimulating FGF2 signaling (Zhang et al., 2001). Since FGF2-knockout mice have reduced bone mass and bone formation (Montero et al., 2000), we speculate that a downregulation of *GPC1* may affect bone development by interfering with FGF2 signaling. Furthermore, glypican-1 has also been demonstrated to bind bone morphogenetic protein 2 (BMP2) and inhibit its functional activity in a human cranial osteogenesis model (Dwivedi et al., 2013). Therefore, a reduction in *GPC1* expression could affect bone formation by altering BMP2 signaling activities.

#### Upregulated Genes *CCN5, TGFBR3*, and DCN

CCN5, also known as WNT1 inducible signaling pathway protein 2 (*WISP-2*) or Connective Tissue Growth Factor-Like Protein (*CTGF-L*), is a member of the connective tissue growth factor/cysteine-rich 61/nephroblastoma overexpressed (CCN) family. The CCN family of proteins play an important role during embryonic development, wound healing, injury repair, angiogenesis and fibrosis (Grünberg et al., 2018). CCN5 is a repressor of the transforming growth factor β (TGF-β) signaling pathway by restricting transcription of the TGF-βRII gene (Sabbah et al., 2011) thereby exerting anti-fibrotic effects. In addition, CCN5 induces canonical WNT pathway which is important for the determination of the fate of mesenchymal stem cells (MSC) (Grünberg et al., 2018). *CCN5* is expressed in primary human osteoblasts cultures (Kumar et al., 1999) and in mature murine osteocytes (Kawaki et al., 2011). Supplementation of recombinant CCN5 protein improved osteoblast mineralization and upregulated the expression of osteogenic genes osterix, alkaline phosphatase and bone sialoprotein *in vitro* (Kawaki et al., 2011). In addition, the gene expression of *CCN5* increases during terminal differentiation of chondrocytes (Kawaki et al., 2008). These collectively suggest an anabolic effect of CCN5 on bone formation.

*TGFBR3* encodes the TGF-β type III receptor, also known as betagylcan. Although betaglycan lacks signaling capacity, it acts as a coreceptor and potentiates the binding of TGF-β ligands to the TGF-β type II receptor, therefore enhancing the TGF-β signaling pathway (Villarreal et al., 2016). Betaglycan is required for inducing osteoblast differentiation and promoting osteogenesis (Hill et al., 2015; Cook et al., 2019).

Therefore, the upregulation of *CCN5* and *TGFBR3* may be counterintuitive but perhaps reflect a feedback mechanism and attempt of *MBTPS2*-OI cells to boost bone health.

Decorin, encoded by *DCN*, is a proteoglycan representing a common component of connective tissue and extracellular matrix. It is composed of a protein core with a single covalently attached glycosaminoglycan (GAG) chain consisting of either chondroitin sulfate in mineralized matrices of bone and dentine or dermatan sulfate in soft connective tissues such as skin and ligament (Waddington et al., 2003). Decorin interacts with collagen fibers with high affinity and regulates collagen fibrillogenesis, thereby playing a crucial role in maintaining the structural and biomechanical properties of the connective tissue (Danielson et al., 1997; Keene et al., 2000; Zhang et al., 2006). Hence, changes in decorin expression may have an impact on bone strength and function.

## Conclusion

Few OASIS-dependent genes are suppressed in *MBTPS2*-OI, while BBF2H7- and ATF6-dependent genes are comparable between OI and IFAP/KFSD patients and control fibroblasts. Importantly, we identified genes involved in cartilage physiology that are differentially expressed in *MBTPS2-*OI but not in *MBTPS2*-IFAP/KFSD fibroblasts.

In this study, we identified gene expression changes in response to pathogenic variants in *MBTPS2* causing either OI or IFAP/KFSD through RNA-sequencing-based transcriptome profiling of fibroblasts obtained from patients and healthy donors. The downregulation of SREBP-dependent genes is stronger in OI than in IFAP/KFSD compared to controls, with consistent changes in the relative abundance of fatty acids in *MBTPS2*-OI fibroblasts *in vitro*. Furthermore, in *MBTPS2*-OI fibroblasts we identified changes in the expression of genes involved in bone and cartilage development, namely *CHST3* and *PAPSS2* that are regulated by OASIS, and *VEGFA*, *ADAMTS12* and *DKK1*. These *in vitro* findings generate new insights and open up new hypotheses on mechanisms that could contribute to and/or counter act the disease progression in OI. It is therefore important to promote OMICS studies in other genetic forms of OI in order to facilitate the discovery of the manifold players in the disease pathology that could be part of a common pathomechanism of OI, such as the Dickkopf family of Wnt signaling antagonisers. In a near future, the establishment of relevant *in vitro* and *in vivo* models of bone and cartilage will allow us to test these new hypotheses in order to develop OI-centric therapeutic strategies.

### Study Limitations

The main limitations of our study arise from the rarity of the genetic disorders caused by *MBTPS2* pathogenic variants and the difficulty in obtaining the relevant biological samples for molecular characterization. In this study the number of biological replicates available for *MBTPS2*-OI and IFAP/KFSD was small and only primary fibroblasts could be obtained from minimally invasive skin punch biopsies. This has limited the ability to detect alterations in physiological processes of bone and cartilage that affect the development of osteoblasts and chondrocytes, such as those regulated by the S2P substrates OASIS and BBF2H7 as discussed above.

A further layer of complexity arises from the wide range of age and variability in the genetic background of our cohort of patients. Therefore, this study has adopted a *p*-Value of < 0.01 without correction for multiple testing in order to discover differentially expressed genes with biologically meaningful functions that may contribute to the disease pathogenesis of OI. Nevertheless, the data obtained has allowed us to generate new hypotheses that we will test in relevant disease models in the future.

## Data Availability Statement

The datasets presented in this study can be found in online repositories. The names of the repository/repositories and accession number(s) can be found below: https://www.ebi.ac.uk/ena, PRJEB42767.

## Ethics Statement

The studies involving human participants were reviewed and approved by the Swiss Ethics Committee (KEK-ZH-Nr. 2019-00811). Written informed consent to participate in this study was provided by the participants’ legal guardian/next of kin.

## Author Contributions

CG, MR, UL, LO, and PJL contributed to the conceptualization of the project. SM, PJL, UL, and LO contributed to transcriptomics data analyses and validation. TN and PJL contributed to immunofluorescence and electron microscopy analyses. PS, MP, and MH contributed to fatty acid analyses. C-DL and DH contributed to sterol analyses. SM, PJL, CG, LO, MH, and C-DL contributed to the manuscript preparation. All authors contributed to the review of the manuscript.

## Conflict of Interest

The authors declare that the research was conducted in the absence of any commercial or financial relationships that could be construed as a potential conflict of interest.
