# Supplementary material for: Omics Profiling of S2P Mutant Fibroblasts as a Mean to Unravel the Pathomechanism and Molecular Signatures of X-Linked MBTPS2 Osteogenesis Imperfecta
Source: Front Genet. 2021 May 21;12:662751. doi: 10.3389/fgene.2021.662751 (PMC8176293; doi:10.3389/fgene.2021.662751)

Supplementary Figure S1. Venn diagrams summarizing the overlap of DEGs between OI versus controls and IFAP/KFSD versus controls.

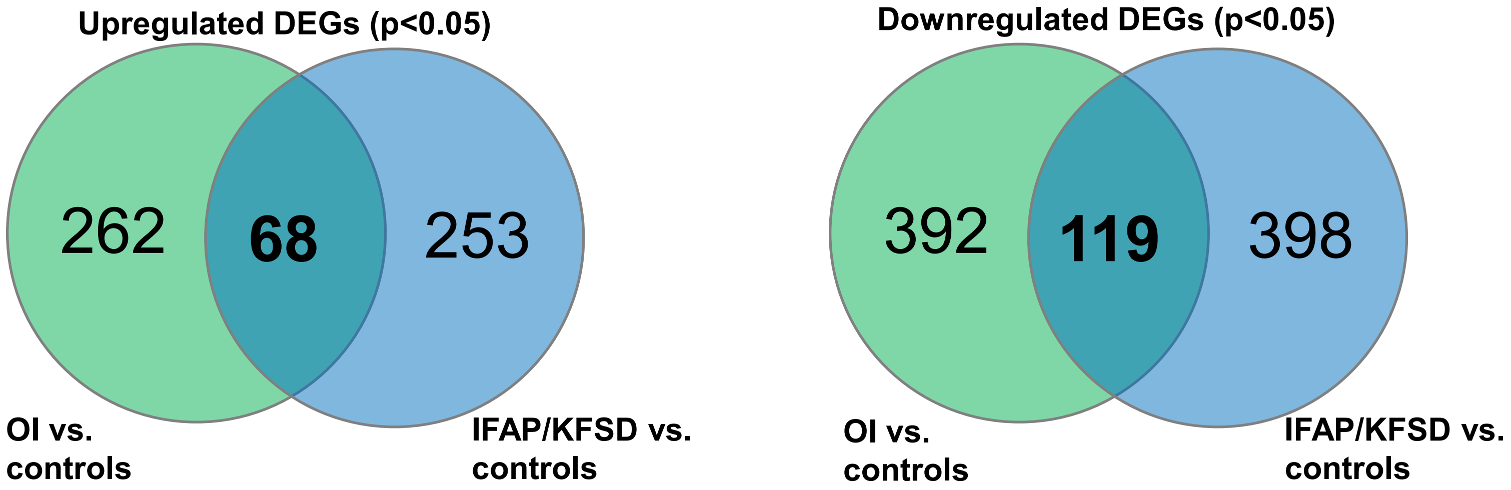

Supplementary Figure S2. Principal Component Analysis biplot of PC1 and PC2 is depicted to visualize the degree of similarities among the gene expression profiles of control and patient fibroblasts at basal conditions (nt) and 24 hours after thapsigargin (TG) treatment to induce ER stress in the top panel. The proportion of variance explained by each PC is shown in the screeplot in the bottom panel.

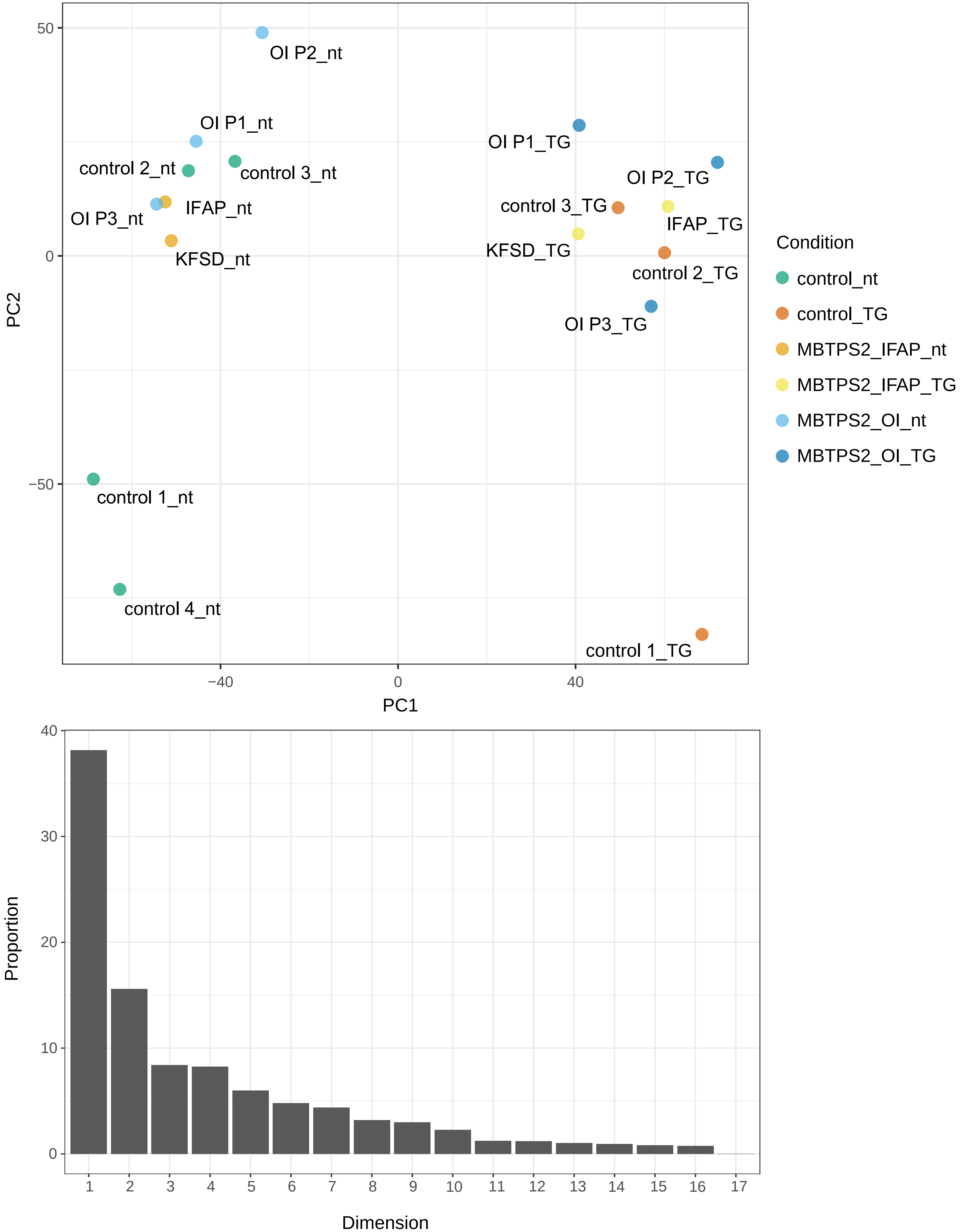

Supplementary Figure S3. The correlation of each sample to all other samples was analysed by Pearson's correlation coefficient.

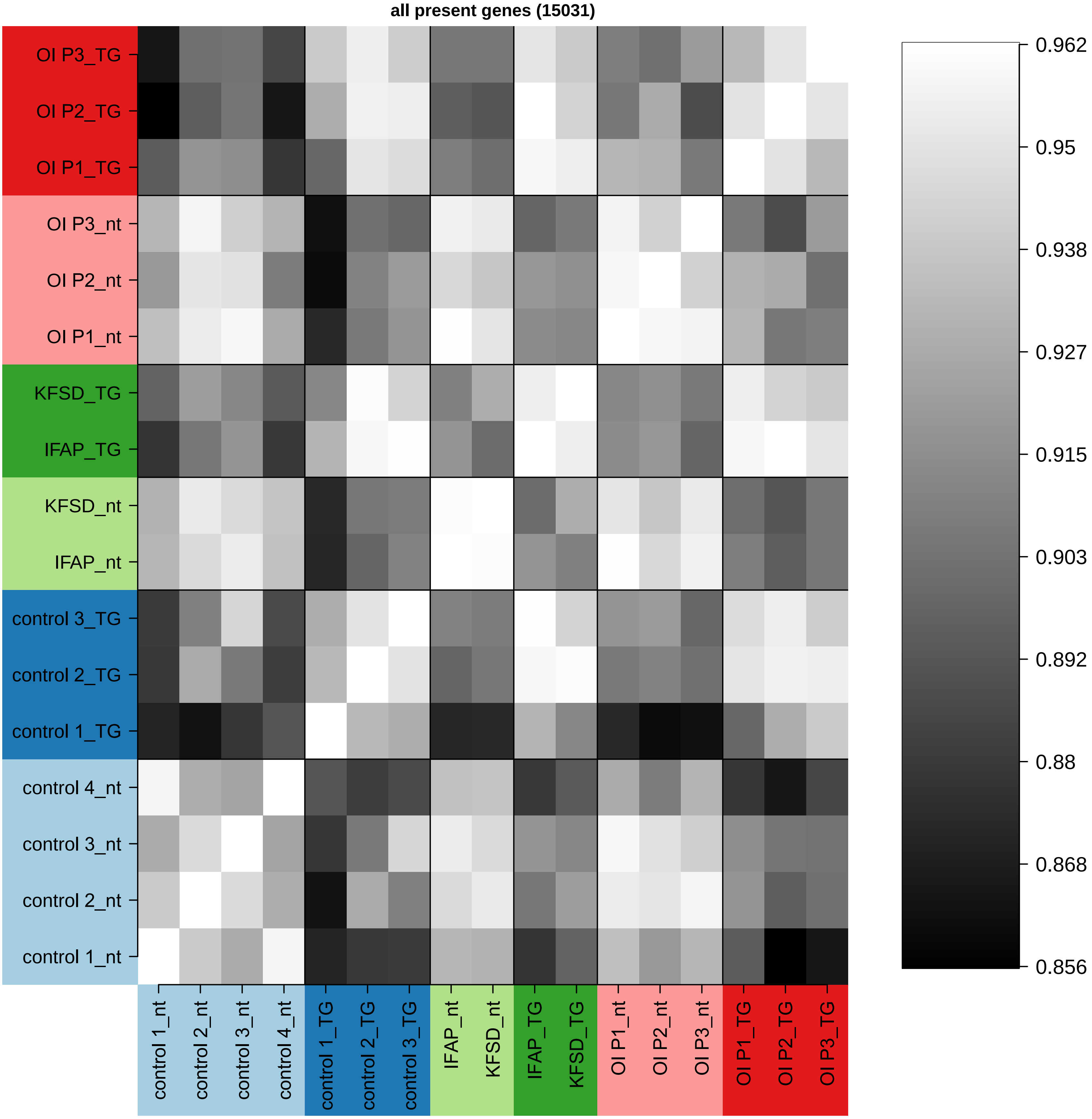

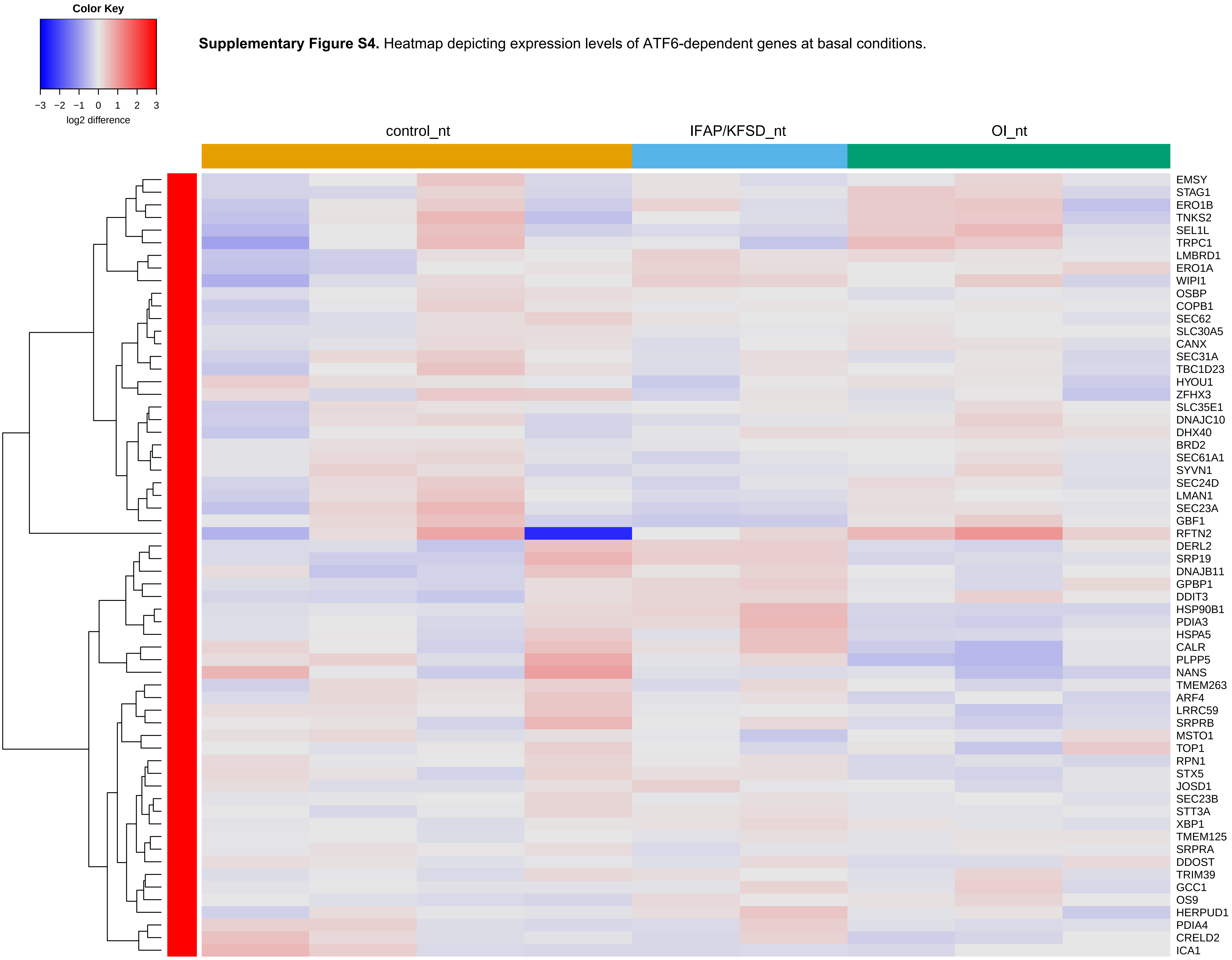

Supplementary Figure S4. Heatmap depicting expression levels of ATF6-dependent genes at basal conditions.

Supplementary Figure S5. Heatmap depicting expression levels of OASIS-dependent genes at basal conditions.

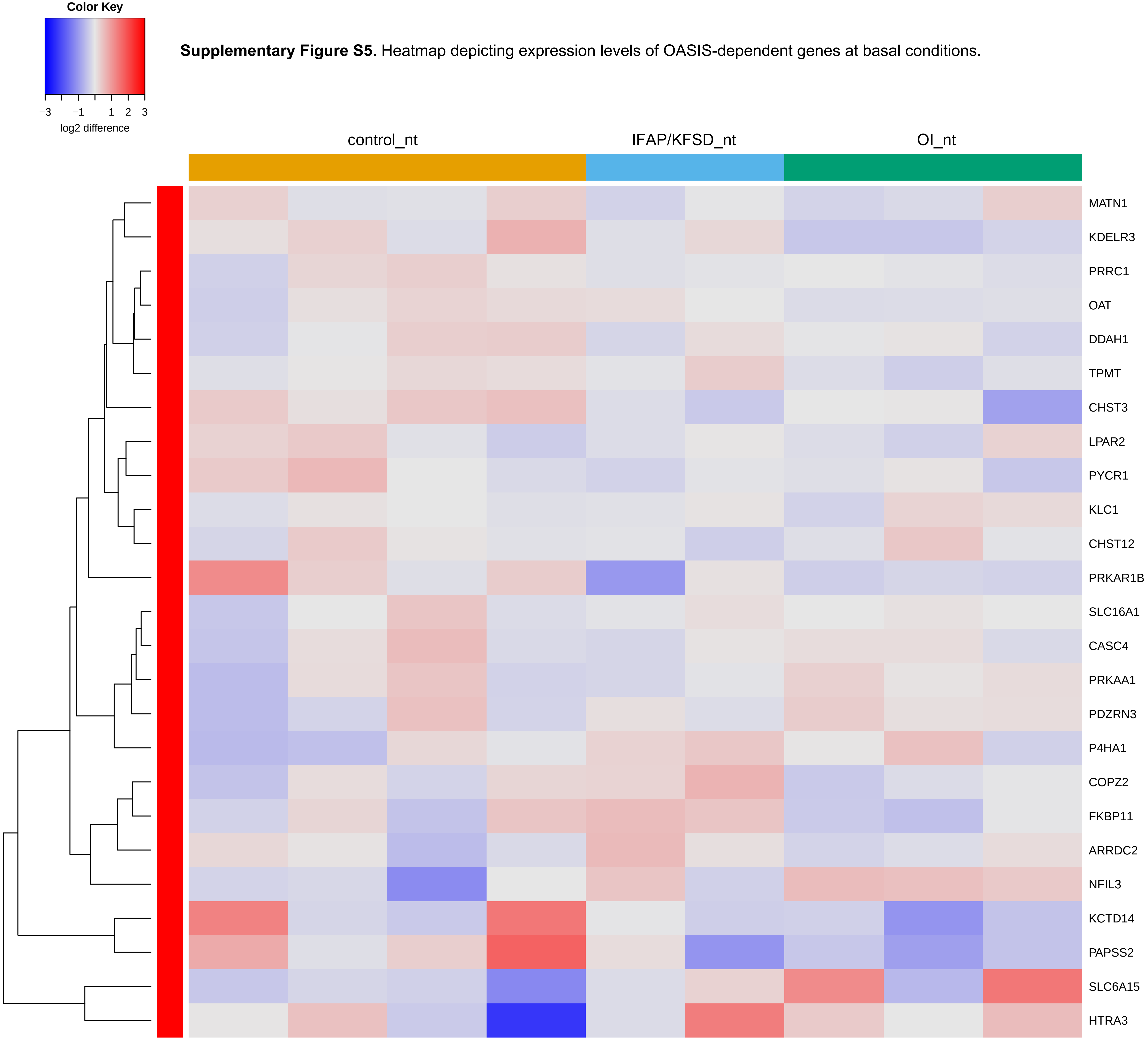

Supplementary Figure S6. Heatmap depicting expression levels of BBF2H7-dependent genes at basal conditions.

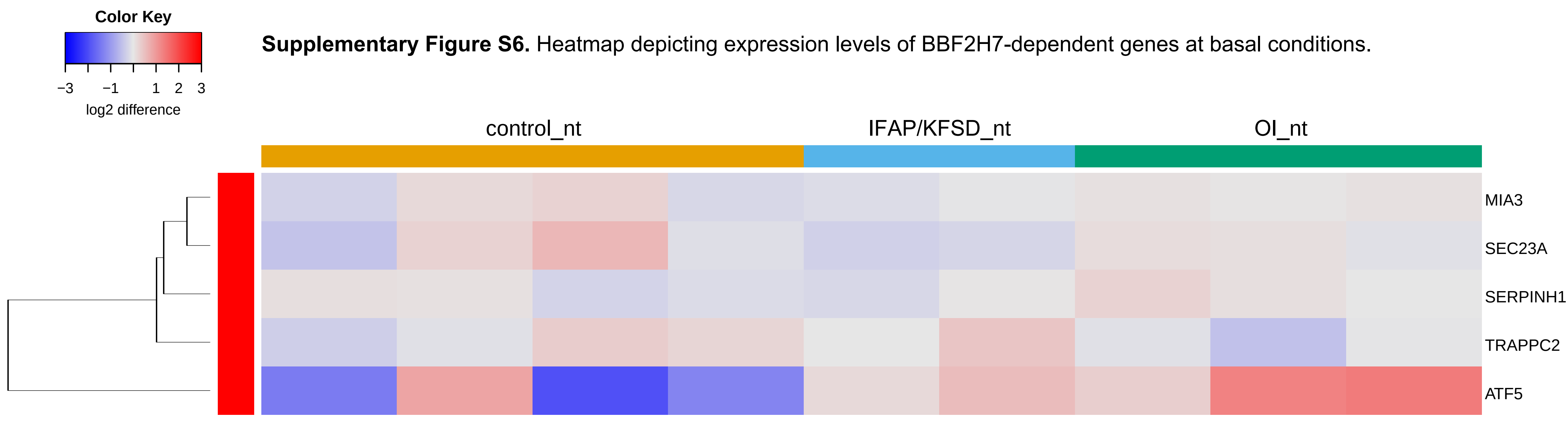

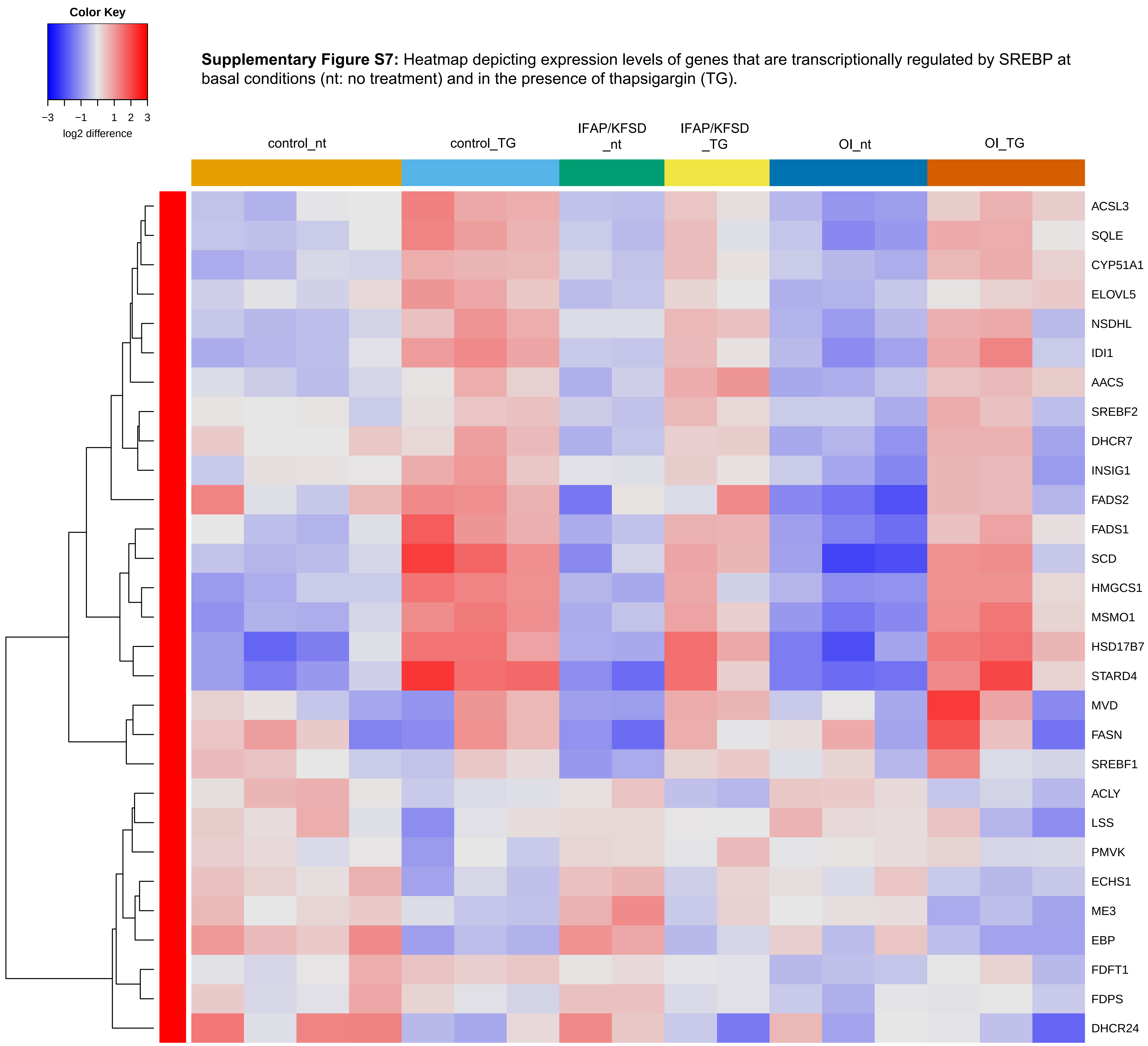

Supplementary Figure S7: Heatmap depicting expression levels of genes that are transcriptionally regulated by SREBP at basal conditions (nt: no treatment) and in the presence of thapsigargin (TG).

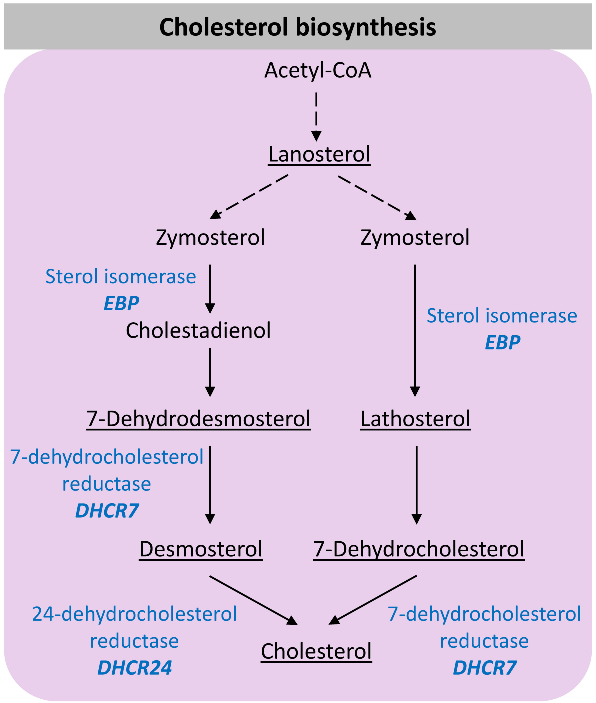

Supplementary Figure S8. A simplified diagram of cholesterol biosynthesis is depicted. The cellular content of sterol metabolites was determined by GC-MS/MS and expressed as ratios. Four independent measurements were performed per subject, and t-tests were performed for statistical analysis. Data are expressed as mean ± SEM.

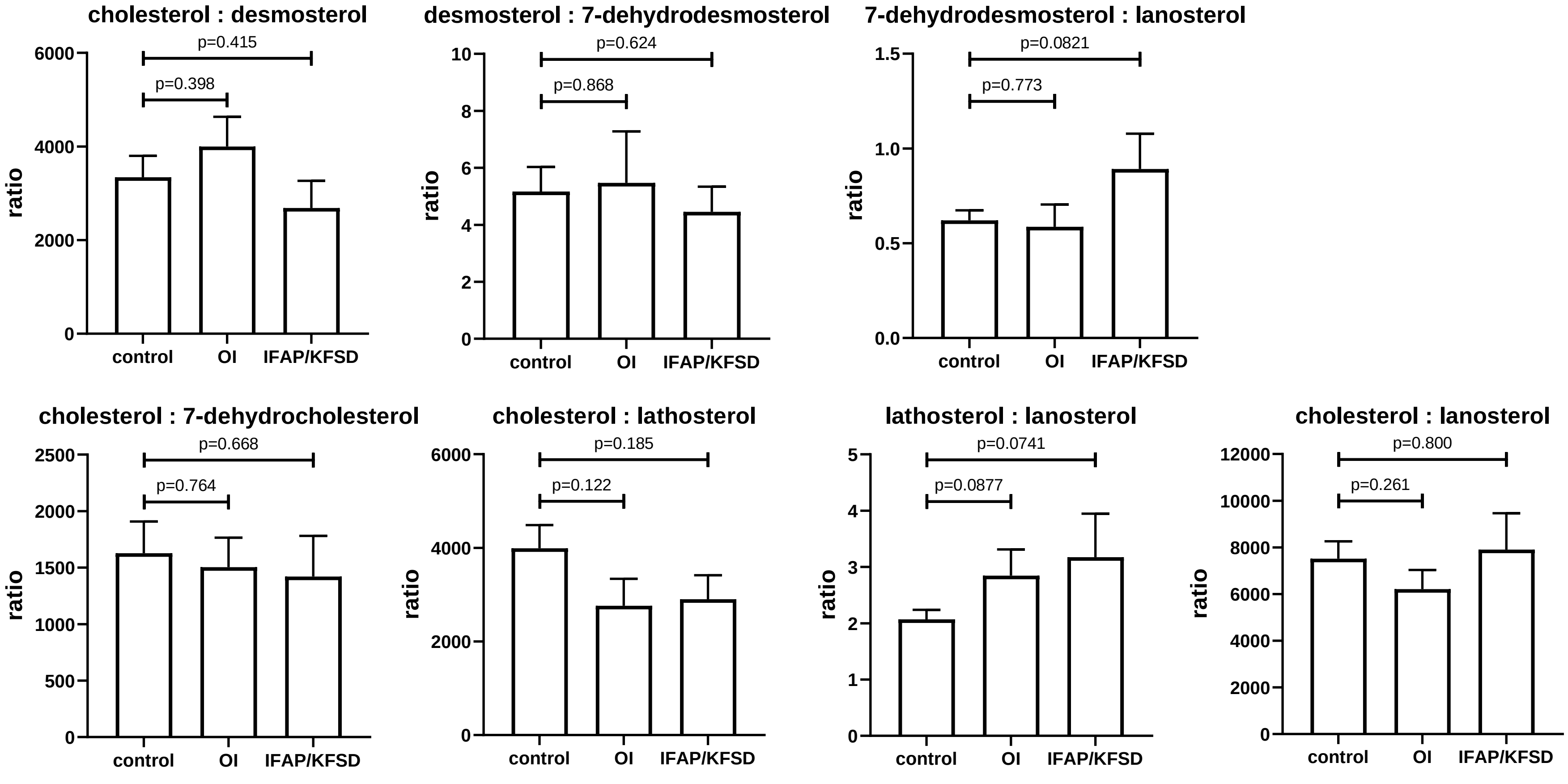

Supplement: Supplementary file 1 [file Data_Sheet_1.PDF]
